# Quantifying the extent of morphological homoplasy: A phylogenetic analysis of 490 characters in *Drosophila*

**DOI:** 10.1002/evl3.115

**Published:** 2019-04-22

**Authors:** Sinan Al Sayad, Amir Yassin

**Affiliations:** ^1^ Institut Systématique Evolution Biodiversité (ISYEB) Centre National de la Recherche Scientifique, MNHN, Sorbonne Université, EPHE 57 rue Cuvier, CP 50, 75005 Paris France

**Keywords:** Character conceptualization, character state coding, developmental hourglass, genitalia, maximum parsimony, recurrent evolution

## Abstract

Homoplasy is a fundamental phenomenon in evolutionary biology but an appraisal of its extent at the morphological level is still lacking. Here, we analyzed the evolution of 490 morphological characters conceptualized among 56 drosophilid species. We found that two thirds of morphological changes were homoplastic and that the level of homoplasy depended on the stage of development and the type of the organ, with the adult terminalia being the least homoplastic. In spite of its predominance at the character change level, homoplasy accounts for only ∼13% of between species similarities in pairwise comparisons. These results provide empirical insights on the limits of morphological changes and the frequency of recurrent evolution.

**Impact Summary**

Is morphological evolution limited, with life being constrained to a small area in the space of all possible forms? Or is it endless, as has been postulated by Darwin 150 years ago? The repeated origin of similar traits in phylogenetically distant lineages, known as homoplasy, challenges Darwin's view but we still lack empirical appreciation of the extent of this phenomenon. Analysis of the evolution of a large set of morphological traits in different developmental stages among 56 fly species revealed that two thirds of morphological changes were homoplastic. Homoplasy was also more frequent in juvenile stages than in adults who showed the highest morphological diversity. Although these findings support the prevalence of homoplasy, they also show that opportunities for the origin of new forms are still higher than it has recently been suggested.

*“Although new and important modifications may not arise from reversion and analogous variation, such modifications will add to the beautiful and harmonious diversity of nature.”*

(Darwin 1859)

Homoplasy, that is, the independent origin of similar character states between distant taxa, is widespread in the living world (Wake et al. 2011), but an appraisal of its extent and underlying mechanisms remains lacking. Some authors argue that homoplasy is so ubiquitous that it is evidence for the limitation and predictability of evolution (Conway Morris 2008; McGhee 2011; Blount et al. 2018). Others, however, caution against this view due to possible experimental and character selection biases (Powell and Mariscal 2015; Stayton 2015). Indeed, whereas long lists of homoplastic examples have been compiled (e.g., Martin and Orgogozo 2013), equivalent lists of nonhomoplastic, that is, apomorphic, states have rarely been made. An account of the diversity of as many character states as possible in clades with well‐defined phylogenies is therefore strongly needed.

Characters are qualities attributed to delimited structures. For molecular data, the structures are nucleotides or amino acid residues at a well‐defined spatial position in a sequence, whereas the qualities are the biochemical compositions of these nucleotides and amino acids. Categorical values with no intermediates could be attributed to these qualities, that is, 4 for nucleotides and 20 for amino acids. For morphological traits, on the other hand, such character conceptualization and categorical coding are difficult (Vogt et al. 2010). In addition, morphological traits are usually stored in the context of phylogenetic analyses wherein autapomorphic states, that is, unique, novel states existing in only a single taxon or species, are often intentionally omitted when parsimony, the predominant approach, is used for tree reconstruction (Bryant 1995; Lewis 2001). This omission could lead to strong biases in estimating homoplasy since homoplasy is time‐independent, whereas synapomorphies, that is, shared, commonly inherited derived states, reflect a particular case of evolutionary stasis wherein a state is maintained with minimal changes for long periods of time.

Here, we address the question of morphological homoplasy in *Drosophila*. One hundred years of genetics and developmental biology research have made this fly one of the best‐understood animals at the morphological level. Annotated genome sequences for multiple species are available and links between genetic mutations and morphological aberrations are curated in accessible online databases such as Flybase (www.flybase.org). Besides the availability of unique genetic toolkits, these resources have made the genus ideal for studies aiming to trace morphological homoplasy between species to their molecular underpinnings (e.g., Wittkopp et al. 2003b; Prud'homme et al. 2006; Kagesawa et al. 2008; Tanaka et al. 2009; Frankel et al. 2012; Signor et al. 2016; Yassin et al. 2016a, b). From two major taxonomic references, we conceptualized 490 morphological characters among 56 drosophilid species. By analyzing the evolution of these traits on a molecularly inferred phylogeny, we were able to quantify the extent of morphological homoplasy in this important clade.

## Materials and Methods

### TAXON SAMPLING

**Figure 1:**
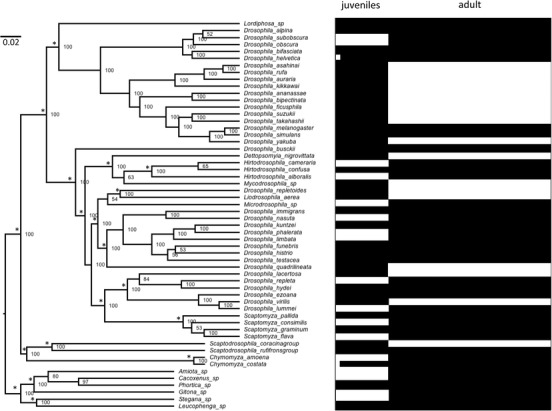
Bayesian consensus phylognetic relationships of the 56 sampled drosophilid species (left) and the extent of coded characters (black) and missing data (white) (right). Values above nodes refer to posterior probability estimates (in percent). Asterisks (*) indicate clades that have been a priori constrained (see text).

We selected 56 drosophilid species for which molecular sequences were available in GenBank and full morphological descriptions could be obtained from two major taxonomic books, Okada's (1968) *Systematic Study of the Early Stages of Drosophilidae* and Bächli et al.’s (2004) *The Drosophilidae (Diptera) of Fennoscandia and Denmark* (Table S1). These books represent two of the most explicit standardized and illustrated descriptions for drosophilids juveniles and adults. We selected species from the main drosophilid clades, that is, the subfamily Steganinae with its two tribes the Steganini and the Gitonini and the subfamily Drosophilinae with its two tribes the Colocasiomyini and the Drosophilini, following Yassin's (2013) classification scheme (see O'Grady and DeSalle 2018 for the current status of drosophilids phylogenetics). The 56 species belonged to 15 genera, including four *Drosophila* subgenera (namely *Sophophora*, *Dorsilopha*, *Drosophila*, and *Siphlodora*). More than one member represented some species groups, e.g., the *melanogaster*, *obscura*, and *quinaria* groups, hence encompassing shallow and profound phylogenetic depths. Some species were treated as “name holder,” having composite DNA sequences or grouping juvenile data from Okada (1968) and adult data from Bächli et al. (2004), such as *Leucophenga* sp, from knowledge or presumption of monophyletic relationships (Table S1; Fig. 1). For morphological data, only 21 species had data from both taxonomic monographs (Table S1; Fig. 1).

### MOLECULAR PHYLOGENETIC TREE

In order to define the phylogenetic relationships of species that were not included in Yassin's (2013) family‐wide phylogenetic revision, we obtained molecular sequences from GenBank for the 56 taxa for one mitochondrial (*COII*) and four nuclear genes (*28S rRNA*, *Adh*, *Amyrel*, and *Gpdh*; Table S1). For each gene, DNA sequences were aligned using the Muscle program (Edgar 2004) with default parameters as implemented by the MEGA7 software package (Kumar et al. 2016). The alignments were then concatenated in a single nexus file (Supporting Information S1). MEGA7 was also used to infer the best DNA substitution model for each gene. For the five genes, the GTR+G+I model had the lowest Akaike Information Criterion (AIC) value and thus was chosen for phylogenetic inference.

Phylogenetic analysis was conducted using MrBayes ver. 3.2. (Ronquist et al. 2012). We used Yassin's (2013) phylogenetic classification as a topological constraint prior given its larger taxonomic and gene sampling. Because the GTR+G+I model was suggested for all genes, we tested unpartitioned model versus partitioned model strategies using stepping‐stone sampling (Xie et al. 2011) implemented in MrBayes. For each strategy, we conducted two simultaneous runs of 1,000,000 generations with sampling every 100 generations, and considered a value of average SD of ≤0.01 an appropriate indicator for convergence between the two runs. Unpartitioned model had a higher marginal likelihood value than partitioned model (–42,712 vs. –42,804). We also used stepping‐stone sampling to test for various clock models using the same run conditions of the unpartitioned data. Relaxed clock models had better marginal likelihood values (TK02 = –42,657 and IGR = –42,658) than the strict (–42,684) and no clock (–42,712). Consequently, we chose the unpartitioned, relaxed clock under the TK02 model. For the trees to be used in subsequent homoplasy analyses in this paper, we performed two simultaneous runs of 2,000,000 generations with sampling every 100 generations for the unpartitioned matrix under the TK02 relaxed clock model (Supporting Information S1). However, we stopped the runs after 1,004,000 generations since an average SD of 0.006 was attained. We then estimated the consensus tree after a burning period of 25% of the 20,086 sampled trees (Fig. 1). In subsequent analyses, we rerooted the consensus and sampled trees by considering the subfamily Steganinae a sister outgroup to the remaining species following the general consensus in drosophilid systematics (Yassin 2013; O'Grady and DeSalle 2018).

### MORPHOLOGICAL CHARACTER CONCEPTUALIZATION

The taxonomic description of specimens consists of a laconic style wherein the name of an anatomical structure is followed by a quality. The same quality (e.g., color) could be attributed to multiple structures (e.g., head, legs, etc.). The same structure (e.g., the aedeagus) could hold multiple qualities (e.g., size, shape, texture, etc.). The same structure bearing the same quality but at different developmental stage (e.g., the number of teeth of the mouth hook of the cephalopharyngeal skeleton in the three larval instars) is conceptualized as a separate character for each stage. Subtle differences in the same quality could lead to the conceptualization of multiple characters. For example, “pleura yellowish” or “pleura with three dark stripes” stem from the same quality, that is, color, but each describes a different character, that is, pigmentation and color pattern, respectively.

### MORPHOLOGICAL CHARACTER STATE CODING

**Figure 2:**
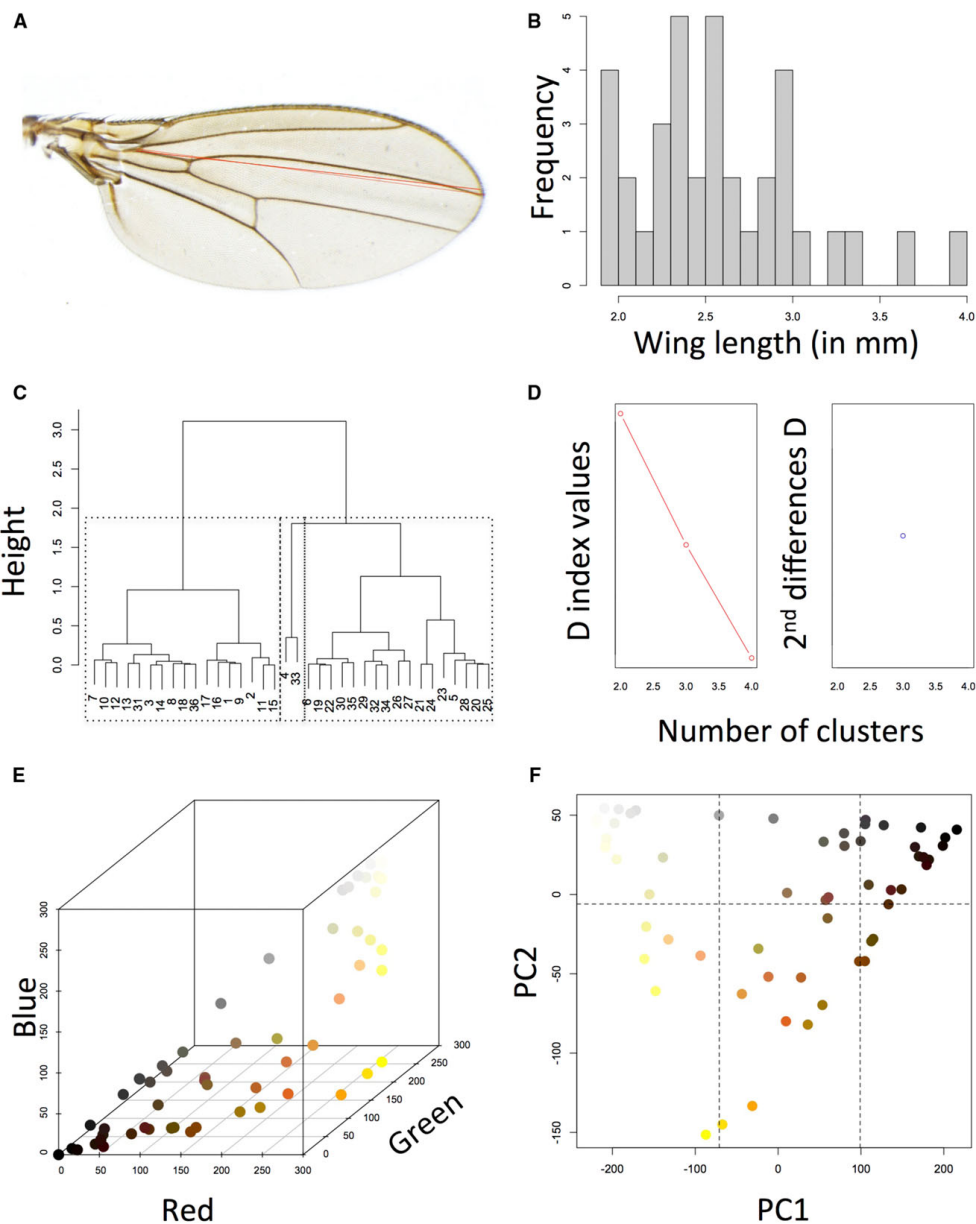
Coding numerical and color traits. (A–D) An example for coding wing length. (A) Definition of the wing length character. (B) Histogram showing the distribution of wing length among the sampled taxa. (C) Hierarchical cluster analysis of wing length values. (D) NBClust estimation of 3 as the optimal number of clusters, delimited with dotted lines in (C). (E) Each of the 60 colors present in the literature was translated into its RGB values. (F) PCA of RGB values followed by cluster analyses on the first two PC axes identified six clusters or states (delimited with dotted lines).

Coding refers to how different values of the same quality of a character in each species could be categorized so that diversity between species and state transformation during evolution could be inferred. We opted for discrete coding, because of its long tradition in the phylogenetic literature and its ability to summarize different types of descriptions such as binary, verbal, and numerical (Supporting Information S2 and S3). Below is how we proceeded for coding the different types of traits:

(1) Numerical descriptions: Numerical values such as lengths, widths, counts (e.g., bristles), and indices were directly obtained for each taxon. When the range over multiple specimens was recorded, we used the average between the extremes as the summarizing value. We then used the NBClust package in R (Charrad et al. 2014) to estimate the optimal number of clusters for each character using Euclidean distance and Ward's method. Values belonging to the same cluster were then attributed the same code in the data matrix. Figure 2A–D shows an example for the coding of a single numerical character (wing length).

(2) Verbal descriptions: Qualities such as “large,” “fusiform,” “concave,” “divergent,” and so on were directly coded when only few distinct values were given (e.g., “concave” vs. “convex”). When illustrations were present, we used ImageJ package (Abramoff et al. 2004) to estimate lengths, angles, and areas for characters for which qualities were slightly ambiguous (e.g., “elliptical” vs. “ovoid,” “subequal” vs. “slightly equal,” etc.). The numerical values obtained from ImageJ were then analyzed using NBClust in R to define the number of optimal states.
(3) Color descriptions: We found 60 verbal descriptions of colors in the two books such as “yellow,” “slightly grayish,” “brownish black,” “reddish brown,” and so on. We translated these descriptions into corresponding RGB values (Table S2, Fig. 2E). Since color perception is highly subjective, each of us produced an independent RGB table for the same descriptions. We then asked a colleague with an experience in *Drosophila* pigmentation to select for the most likely values in cases of disagreement. We conducted a Principal Component Analysis (PCA) on the RGB values, and projection of the two first axes identified a triangular distribution (Fig. 2F) corresponding to the three main *Drosophila* pigments, that is, black and brown melanin, yellow NADA sclerotin, and white NBAD sclerotin (Wittkopp et al. 2003a). We analyzed each of these axes using NBClust and identified six states for colors that were used for all color characters.
(4) Pattern descriptions: Patterns describe the spatial distribution of a quality within a structure. We encountered several pattern descriptions such as trichomes on the larval abdomen, tracheae of the anterior spiracles of the pupa, tubercules on the posterior spiracles of the puparia, pilosity on the genitalia, pigmentation of adult tergites, and so on. For larval trichomes, we followed Okada's (1968) categories. For pupal tracheae and tubercules, we followed a reductive coding, wherein each structure was reduced to its components and qualities of each component coded separately (e.g., tracheae were separated into basals, pseudobasals, centrals, pseudocentrals, and marginal). For pigmentation patterns on adult tergites, a trait that has intensively been studied in *Drosophila* evo‐devo research (Massey and Wittkopp 2016), we divided each tergite into distinct spatial regions (Figs. S1–S7) and coded the colors as described above.

Missing data corresponded to either values that were not given for all taxa or those that are attributed to structures not present in all taxa. For example, steganine eggs lack filaments (Character 7). They were then attributed a missing data sign (‘?’) in all characters describing filaments number and shape in other species whose eggs have filaments (Characters 8–10).

The biological significance of our statistical delimitation remains to be clarified since the genetic basis of only a few morphological changes in drosophilids have yet been determined in evo‐devo research (Table 1). From this literature, we found that our approach has led to “state lumping”, that is, coding genetically different states into similar categories, for the number of hypandrial bristles (Character 394). This character was statistically coded as having three states with state 0 bearing 0 to 3 pairs. However, the transition from 1 pair to 0 bristles between a pair of *Drosophila* species has a strong genetic component (Nagy et al. 2018). Consequently, we used the biological information to recode 0 bristles as a fourth state for this character.

**Table 1:** Examples of morphological characters studied in *Drosophila* evo‐devo studies

No.	Character	References	This study
1	Egg filaments	(Kagesawa et al. 2008; Osterfield et al. 2015)	Yes
2	Egg dorsal ridge	(Niepielko and Yakoby 2014)	Yes
3	Larval hooklets	(Frankel et al. 2012)	Yes
4	Puparium color	(Ahmed‐Braimah and Sweigart 2015)	Yes
5	Head shape	(Arif et al. 2013a)	Yes
6	Dorsocentral bristles	(Marcellini and Simpson 2006)	Yes
7	Protarsal bristles (sex combs, brushes)	(Tanaka et al. 2009; Atallah et al. 2014b; Rice et al. 2018)	Yes
8	Mesofemoral bristles	(Arif et al. 2013b)	NO
9	Wing color patterns	(Prud'homme et al. 2006; Werner et al. 2012; Arnoult et al. 2013)	Yes
10	Abdomen tergal pigmentation	(Wittkopp et al. 2003b; Jeong et al. 2008; Rebeiz et al. 2009; Signor et al. 2016; Yassin et al. 2016a,b; Grover et al. 2018)	Yes
11	Male abdominal muscles	(Orgogozo et al. 2007)	NO
12	Epandrial posterior lobe	(Glassford et al. 2015; Tanaka et al. 2015)	Yes
13	Hypandrial bristles	(Nagy et al. 2018)	Yes
14	Aedeagal basal shape	(Peluffo et al. 2015)	Yes
15	Ovariole number	(Orgogozo et al. 2006; Green and Extavour 2012)	No
16	Oviscapt shape	(Atallah et al. 2014a)	Yes

### QUANTIFYING ENSEMBLE HOMOPLASY

Several comparative phylogenetic measurements and tests for homoplasy have recently been developed (Speed and Arbuckle 2017). However, for discrete data, traditional cladistics measurements such as the ensemble consistency (CI) and homoplasy (HI) indices (Kluge and Farris 1969) and the retention indices (RI, Farris 1989) remain the only developed statistics (Speed and Arbuckle 2017). We thus used PAUP* version 4.0a (Swofford 2002) to estimate these indices for the whole data set, using the Describe Tree command under both the ACCTRAN and DELTRAN models and all characters were unordered. To account for topological uncertainties, the indices averaged over the 20,086 sampled trees from the two simultaneous MrBayes runs (Supporting Information S3). Given the strong topological constraints we applied (see above), we averaged indices over all sampled trees and not only on those retained after burnin.

### COUNTING HOMOPLASTIC STATES

We used the PAUP* to produce the list of character changes (DescribeTrees/chgList) as well as to infer ancestral states (DescribeTrees/internal) on the consensus Bayesian tree under the ACCTRAN and DELTRAN models. We then parsed the output file to count for each character state the number of times it has been derived and shared between taxa. This led to five possible categories: (1) nonhomoplastic root states, (2) homoplastic root states (i.e., root states that have been secondarily derived), (3) homoplastic derived states, (4) synapomorphic states (i.e., states that have been derived once and are shared by at least two terminal taxa), and (5) autapomorphic states (i.e., states that have been derived once and are specific to a single terminal taxon).

### TESTING THE EFFECTS OF DEVELOPMENTAL STAGES AND BODY ORGANIZATION

A character consistency index (*c_i_*) of 1 indicates that all states are apomorphic, that is, they have been derived once. While a *c_i_* < 1 is a sign of homoplasy in the character, *c_i_* < 1 values cannot be compared between characters because the lower boundary of *c_i_* depends on the number of character states (e.g., being 0.5 for a binary character). The *r_i_* was proposed to correct for this limitation. A character retention index (*r_i_*) value of 1 also indicates apomorphy, but unlike the *c_i_*, *r_i_* cannot be calculated for autapomorphic traits, that is, characters with derived states appearing in only one terminal taxon. Consequently, an *r_i_* of 1 indicates only synapomorphies, that is, commonly inherited derived states among a group of taxa. For each character, we estimated both *c_i_* and *r_i_* using the PAUP* DescribeTrees/diagnose command on the 20,086 sampled Bayesian trees. Averaged values for each character were then used to test for differences in the amount of homoplasy among developmental stages and body parts. For this, characters were classified according to six developmental stages: egg, larva 1, larva 2, larva 3, pupa, and adult, with the adult characters being further classified into five body parts: head, thorax, abdomen, male terminalia, and female terminalia. We also tested the effect of autapomorphies by conducting statistical tests on *r_i_* without autapomorphies or after attributing an *r_i_* value of 1 to autapomorphic characters (hereafter denoted *r_ic_*). We conducted all statistical analyses and model testing using R (www.R-project.org).

### DISTINGUISHING BETWEEN HOMOPLASTIC MECHANISMS

Traditionally, homoplasy is thought to result from three major mechanisms: parallelism, convergence, and reversal. There are confusions on the distinction between parallelism and convergence, with the former meaning similar initial conditions of the homoplastic state between the compared taxa and the latter referring to distinct origins (reviewed in Pearce 2012 and Stayton 2015). Some authors consider the “origin” in developmental terms, for example, changes in similar or different genes for parallelism or convergence, respectively. Others consider the “origin” as the ancestral placement on the morphospace regardless to the underlying developmental mechanism as proposed by Jablonski (cited in Pearce 2012). We follow here Jablonski's definition for its operationality in phylogenetic analyses.

**Figure 3:**
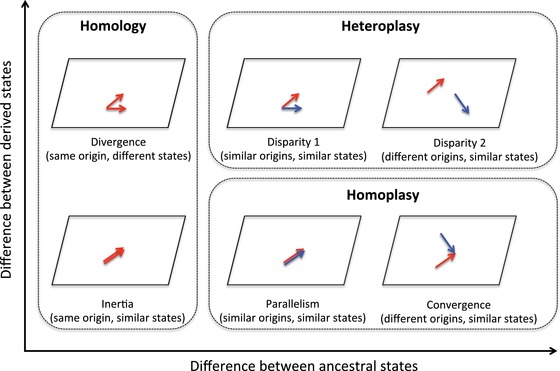
Different categories of trait evolution on the morphospace between pairs of species according to the degree of resemblance between states of the ancestors and the descendants.

We distinguished six possible changes for character spaces in a pair of species depending on the degree of sameness, similarity, and difference of the ancestral and present states in the two species (Fig. 3). We then used PAUP* to infer under the ACCTRAN and DELTRAN models the ancestral states at each internal node. Using a customized perl script we obtained for each taxon for each trait its current state, its original state, and the internal node at which the state changed (Supporting Information S4). For each trait, we conducted all possible pairwise comparisons between species with no missing data and estimated the frequency of each change category (Supporting Information S4).

Unlike parallelism and convergence, reversal does not imply comparisons between taxa, but a comparison between the state present in a taxon with the states present in its ancestors, and consequently this may lead to any of the possible between‐species mechanisms shown in Figure 3. Using a perl script (Supporting Information S5), we compared for each trait the current state of each taxon with that of its ancestors and counted incidences were a reversal (e.g., 0 → 1 → 0) was found. Because the path of each taxon to the root had to be manually entered in the script from PAUP* node labeled cladogram, we conducted these analyses on a single tree, that is, the consensus Bayesian phylogeny.

## Results

### AT LEAST 487 MORPHOLOGICAL CHARACTERS DIFFER BETWEEN DROSOPHILID SPECIES

Thorough reading of the descriptions of the 56 species led to the conceptualization of 490 characters from drosophilids eggs (*N* = 12), larvae1 (*N* = 13), larvae 2 (*N* = 23), larvae 3 (*N* = 26), pupae (*N* = 46), and adults (*N* = 370). Adult characters were also classified into traits on the head (*N* = 78), thorax (*N* = 90), abdomen (*N* = 40), male terminalia (*N* = 150), and female terminalia (*N* = 12). The total number of characters states was 1479, ranging from 1 to 8 with an average of three states per character. Three characters were invariable having a single state, that is, egg color (always white or greyish white), the number of ventral caudal tubercles in the pupa (always 2), and the presence of an apical bristle on the mesotibia (always present).

### TWO THIRDS OF MORPHOLOGICAL CHANGES ARE HOMOPLASTIC

**Figure 4:**
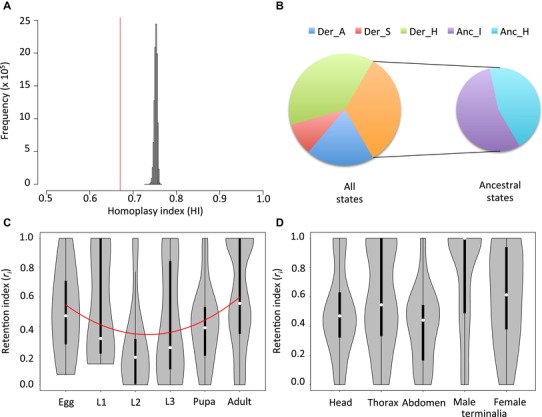
Homoplasy measurements for whole data, states and characters. (A) Histogram of the whole‐data homoplasy index (HI) after 10,000,000 random permutations of taxa over the phylogenetic tree. The empirical value of 0.67 (averaged over 20,086 sampled Bayesian trees) is shown in red and falls outside the random distribution. (B) Proportions of morphological states categorized as derived (Der) and ancestral (Anc) and subcategorized as autapomorphic (A), synapomorphic (S), homoplastic (H) and invariable (I). (C‐D) Violin plots of the retention index (*r_ic_*) with autapomorphic attributed an *r_i_* value of 1 for the 487 morphological characters. Plots are arranged according to (C) the developmental stage and (D) the adult organs. White dots inside the boxes indicate the distribution median. L1, L2 and L3 refer to 1^st^, 2^nd^ and 3^rd^ larval instars, respectively. Polynomial regression suggesting a “developmental hourglass model” is shown as red curve in (C).

Under both the ACCTRAN and DELTRAN models, consistency index (CI) for the whole data set averaged over all 20,086 trees sampled from the two runs of MrBayes was 0.33 (±4.37 × 10^−6^), with its complement, the data homoplasy index (HI), being 0.67 (±4.37 × 10^−6^; Fig. 4A). This means that for a character change in *Drosophila* there is nearly as much as twice a chance that the character will take a recurrent or a preexistent state than a new one. Interestingly, average HI value after 10,000,000 random permutations of taxa over the tree using PAUP* was 0.75, with the empirical value of 0.67 falling outside the random distribution (Fig. 4A). Although this result supports the predominance of morphological homoplasy, it still shows that the potential space for novelties in drosophilids morphology is considerably large.

### ONLY ONE THIRD OF DERIVED CHANGES ARE SHARED

PAUP* was used to infer the ancestral state at the root of the phylogenetic tree for the 490 characters. Under the ACCTRAN model, 220 of the 490 root states have been secondarily derived. Among the 989 derived states, 429 were derived once. Of these, 137 states (∼32%) are shared between at least two taxa, that is, synapomorphic states (Fig. 4B). Under the DELTRAN model, 122 root states were secondarily derived, and 113 of 405 apomorphic states were synapomorphic (∼28%). That means that only one third of states occupying novel positions on the morphospace would remain unchanged for longer evolutionary times. This result cautions against homoplasy quantification from only phylogenetically informative characters, that is, after excluding characters with only autapomorphic derived states, as it is customary in cladistic analyses (Bryant 1995).

### DEVELOPMENTAL CHANGES IN HOMOPLASY SUPPORT A MORPHOLOGICAL ‘HOURGLASS’ MODEL

Homoplasy, as measured by the character retention index with characters with only autopamorphic states attributed an *r_i_* value of 1 (here *r_ic_*), significantly differed among developmental stages (Kruskall–Wallis chi‐squared = 34.013, *df* = 5, *P* = 2.37 × 10^−6^; Table S3; Fig. 4C). The difference formed a concave pattern (polynomial regression: *r_ic_* = 0.755 – 0.245 *D* + 0.037 *D* ^2^, *P* = 0.003, with *D* referring to the developmental stage as egg, larva 1, larva 2, larva 3, pupa, and adult with a unit increment). This quadratic regression had a lower Akaike Information Criterion (AIC) than a linear regression (352.45 vs. 363.20, respectively). The concave pattern, which supports the “developmental hourglass model” (see Discussion below), did not differ when autapomorphic characters were excluded (*r_i_* = 0.588 – 0.201 *D* + 0.030 *D* ^2^, *P* = 0.006). No differences were found between the ACCTRAN and DELTRAN models.

### MALE AND FEMALE TERMINALIA HAVE THE LEAST HOMOPLASTIC CHARACTERS

Homoplasy also differed between adult organs (Kruskall–Wallis chi‐squared = 41.935, *df* = 4, *P* = 1.72 × 10^−8^), with male and female terminalia characters being the least homoplastic compared to the three somatic organs, that is, the head, the thorax, and the abdomen (Wilcox test: W = 10986, *P* = 7.39 × 10^−9^; Table S3; Fig. 4D). This difference did not persist when autapomorphic characters were excluded (Wilcox test: W = 7119, *P* = 0.18). Although on average, male terminalia had higher *r_ic_* than female organs (0.73 vs. 0.61, respectively), the difference was not significant (Wilcox test: W = 1107.5, *P* = 0.158). Exclusion of autapomorphic characters did not lead to significant difference either (Wilcox test: W = 349, *P* = 0.32). This lack of strong divergence between the sexes in terminalia traits supports the hypothesis of genital coevolution for which several lines of evidence have recently been demonstrated in *Drosophila* (Kamimura 2007; Yassin and Orgogozo 2013) and other animals (Yassin 2016). No differences were found between the ACCTRAN and DELTRAN models.

### PARALLELISM AND CONVERGENCE ACCOUNT FOR 13% OF PAIRWISE SPECIFIC SIMILARITIES

**Figure 5:**
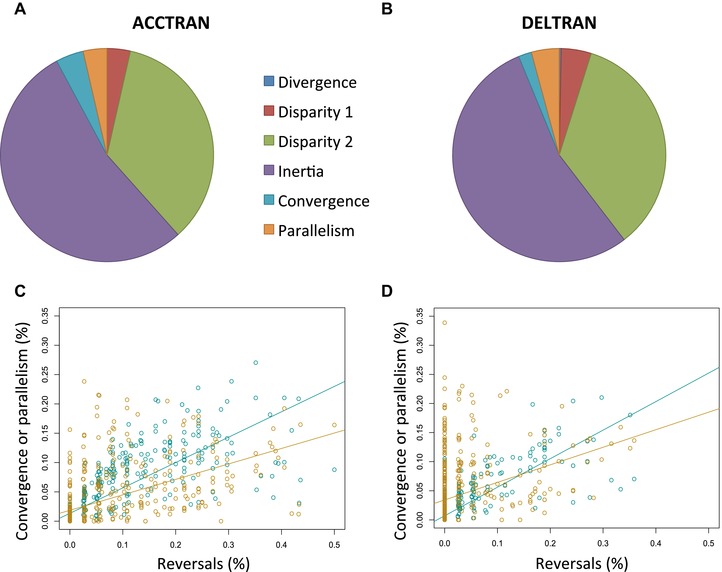
Extent of mechanisms underlying homoplasy in pairwise species comparisons. (A and B) Proportions of different mechanisms of similar and dissimilar states (explained in Fig. 3) averaged over all characters under the ACCTRAN (A) and DELTRAN models (B). (C and D) Correlation between proportion of descendants with a reversal to an ancestral state and the proportion of convergence (dark violet squares) and parallelism (turquoise circles) for each character under the ACCTRAN (C) and DELTRAN (D) models.

Proportions of instances belonging to each of the six possible cases of character changes significantly differed (Table S3, Fig. 5A and B). For similarity between pairs of species, the most frequent case was that of phylogenetic inertia (ACCTRAN = 53.77%, DELTRAN = 54.20%), that is, the two species have a state that derived from the state present in their last common ancestor. Under the ACCTRAN model, this figure was followed by both convergence (4.22%) and parallelism (3.61%), which together indicates that homoplasy does not exceed 13% of cases where two species have similar character states. Under DELTRAN, parallelism (4.22%) exceeded convergence (1.99%), with sum homoplasy counting for 10.3% of species comparisons with similar states. For differences between species, the most frequent case was that of Disparity 2 (ACCTRAN = 34.86%, DELTRAN = 34.72%), wherein different states arose from different ancestral states. This was followed by Disparity 1 wherein the ancestral states were similar but of different origins (ACCTRAN = 3.54%, DELTRAN = 4.56%). No (0.00%) and 94 cases (0.30%) of divergence were found in pairwise comparisons where different states diverge from the same different ancestral state under the ACCTRAN and DELTRAN models, respectively.

### REVERSALS OCCUR IN 7% OF DESCENDANT–ANCESTRAL COMPARISONS

Two hundred sixty‐one (53%) and 363 (74%) characters showed no single occurrence of reversals under the ACCTRAN and DELTRAN reconstructions, respectively. On average, reversals were found in 6.65% and 2.63% of all comparisons between terminal taxa and their ancestors according to the two models. The frequency of reversals correlated with both parallelism and convergence, although its effect on convergence was higher under the ACCTRAN model (*R^2^* = 0.62 and 0.29 for convergence and parallelism, respectively). Similar correlations were also found under the DELTRAN model (*R^2^* = 0.65 and 0.11 for convergence and parallelism, respectively). These results suggest that high incidences of convergence and parallelism could be mostly due to recurrent reversals (Table S3, Fig. 5C and D).

## Discussion

### WHAT IS THE EXTENT OF MORPHOLOGICAL HOMOPLASY?

Our analyses on 490 morphological traits indicate that nearly two thirds of morphological changes are homoplastic. Our ensemble consistency index (CI) estimate of 0.33 approaches that obtained in other cladistics analyses with >300 morphological characters, for example, in Diptera (CI = 0.40, Lambkin et al. 2013), Birds (0.24, Livezey and Zusi 2007), Birds and related Dinosaurs (0.27, Godefroit et al. 2013), Squamates (0.16, Conrad 2008; 0.21, Gauthier et al. 2012), and Placental Mammals (0.20, O'Leary et al. 2013). Our study differs in its narrower taxonomic scale (i.e., all species belonging to the same family) and its quantification of morphological homoplasy on an independently inferred molecular tree. Zou and Zhang (2016) have recently compared O'Leary et al.’s (2013) ∼3,500 morphological traits with a molecular phylogeny and estimated a morphological ensemble CI value of ∼0. 25. Testing whether the potential of morphological innovations is larger in Dipterans (or Insects) than in Vertebrates would require further developments and refinements of the so‐called “giant taxon‐character matrices” (Simões et al. 2017; Laing et al. 2018).

### HOW MANY MORPHOLOGICAL CHARACTERS ARE THERE?

In order to draw general conclusions about the extent of homoplasy, one should be aware about how representative is the studied sample of characters. Information on the total number of morphological traits is lacking, not only because their study often requires profound learning and expertise but also because of the lack of a standard conceptualization approach. To the best of our knowledge, our study represents the largest morphological dataset to be analyzed for the prevalence of homoplasy in an insect. Various *Drosophila* evolutionary developmental studies have investigated some of the traits analyzed here (Table 1). Morphological phylogenetic studies have analyzed 18 (Throckmorton 1962 as reanalyzed by Grimaldi 1990), 217 (Grimaldi 1990), 68 (Hu and Toda 2001), and 37 traits (Yassin 2013). However, it remains difficult to recognize the degree of overlap between these studies because different authors often used different concepts for the same characters and/or different coding approaches.

Vogt et al. (2010) highlighted the need for standardizing morphological terms among disciplines with a morphological character being defined as a quality attributed to a delimited anatomical structure The 490 characters analyzed here corresponded to 140 anatomical structures and nearly seven qualities (size, shape, count including presence/absence, color, pattern, texture, and hardness). Our reliance on the taxonomic literature (with its emphasis on variable traits) and our taxonomic sampling and scope have definitively overlooked counting multiple invariable characters (e.g., the presence/absence of the eyes and the legs, the number of abdominal tergites, etc.). Invariable characters are also omitted in cladistics analyses using parsimony in spite of their relevance in rate estimate in probabilistic phylogenetics (Lewis 2001). On Flybase (as of July 2018), ∼10,000 anatomical terms have been described in *D. melanogaster*, half of which have no descendant term and hence may form the basis for a standard anatomical description. Assuming that those seven qualities are measured for each of the 5,000 structures, the minimal number of morphological characters may be 35,000. This figure largely exceeds (nearly 70 times) our current analysis and strongly reflects the paucity of our understanding of the evolution of morphological structures.

### WHAT ARE THE GENOMIC UNDERPINNINGS OF MORPHOLOGICAL HOMOPLASY?

Our morphological analyses uncovered two interesting genomic evolutionary trends, namely the developmental “hourglass” model and the rapid evolution of sex‐specific traits. The “hourglass model” postulates that intermediate stages of development are evolutionarily less variable than early and later stages (Irie and Kuratani 2014). In the context of our analysis, low variability correlates with high homoplasy, that is, there are higher chances to find similar states among taxa at larval stages than at the egg, pupal or adult stages. The hourglass model was initially proposed based on morphological data in Vertebrates, but its support in insects mainly came from genomic and transcriptomic analyses in *Drosophila* (Cruickshank and Wade 2008; Kalinka et al. 2010; Yassin et al. 2010) and Diptera (Jiménez‐Guri et al. 2013; Schep and Adryan 2013). Our results support the prevalence of the model at the morphological level in an insect but it is important to note that we did not study embryos, since the egg analyzed here is the female gamete with most features such as size, shape, and chorionic specializations (e.g., dorsal appendages, ridges, and sculpture) being determined during oogenesis. Moreover, characters were unevenly distribution on the phylogeny (Fig. 1). Investigating these missing data in future comparative studies should improve our tests of different developmental patterns.

Song and Bucheli (2010) found no significant difference in the phylogenetic signal between genital and non‐genital traits in a meta‐analysis of 41 studies in insects wherein autapomorphic characters were excluded. Similarly, we did not find a difference in the amount of homoplasy between adult genital and non‐genital characters when autapomorphies were also excluded. Nonetheless, including autaopomorphies revealed a sharp difference. Rapidly evolving characters should be richer in autapomorphic states, because such states have either recently appeared or rapidly changed in other lineages. Our finding hence is consistent with previous studies using fewer morphological characters in *Drosophila* (Civetta and Singh 1998) and with recent genome‐wide analyses revealing faster evolution of traits and genes with sex‐limited expression (Haerty et al. 2007; Parsch and Ellegren 2013), most likely due to divergent sexual selection that could ultimately lead to fewer homoplasy.

Ever since the early days of *Drosophila* genetics, a major question has arisen on the relationship between the genetic mutations discovered in *D. melanogaster* and the morphological differences between wild species (Sturtevant 1921). Patterson and Stone (1952) argued that resemblance between morphological differences and *D. melanogaster* mutants might involve homologous or quite different loci, which seems to be the case as unraveled by recent evo‐devo studies (e.g., Frankel et al. 2012, 2016b; Yassin et al. 2016a; Yassin et al. 2016b). On the online database Flybase, the effect of genetic mutations on different anatomical structures is curated, and for each gene in *D. melanogaster* links to its orthologous genes (when present) in other *Drosophila* species are also provided. However, data on the evolutionary differences in these structures are still scattered in the taxonomic literature for the nearly 4,500 drosophilid species. The matrix provided here represents a first attempt to curate these disperse data and future research should complement missing data, adding more taxa and enriching Flybase resources with structures evolution. This endeavor would significantly promote the identification of the genetic underpinnings of morphological changes leading to better character state delimitation. Given the prevalence of the “deep homology” of developmental pathways in animals, such knowledge would ultimately lead to a better understanding of the molecular basis of morphological homoplasy in *Drosophila* and beyond.

Associate Editor: A. Goswami

## Supporting information

Supporting Information

**Table S1**. Phylogenetic classification, data origin and GENBANK accession numbers for taxa sampled in the present study.

**Table S2**. RGB coding and NBClust analysis for the 60 colors described in the taxonomic literature.

**Table S3**. Character evolution statistics (ci=consistency index, ri=retention index, rc=rescaled consistency index, hi = homoplasy index) and proportions of different evolutionary mechanisms for the 490 morphological characters analyzed in the present study.

Supporting Information

Supporting Information

Supporting Information

Supporting Information

Supporting Information

## References

[evl3115-bib-0001] Abramoff, M. D. , P. J. Magalhães , and S. J. Ram 2004 Image processing with Image. J. Biophotonics Int. 11:36–42.

[evl3115-bib-0002] Ahmed‐Braimah, Y. H. , and A. L. Sweigart 2015 A single gene causes an interspecific difference in pigmentation in *Drosophila* . Genetics 200:331–342. 25769982 10.1534/genetics.115.174920 PMC4423374

[evl3115-bib-0003] Arif, S. , M. Hilbrant , C. Hopfen , I. Almudi , M. D. S. Nunes , N. Posnien , L. Kuncheria , et al. 2013a Genetic and developmental analysis of differences in eye and face morphology between *Drosophila simulans* and *Drosophila mauritiana* . Evol. Dev. 15:257–67. 23809700 10.1111/ede.12027 PMC3799016

[evl3115-bib-0004] Arif, S. , S. Murat , I. Almudi , M. D. S. Nunes , D. Bortolamiol‐Becet , N. S. McGregor , J. M. S. Currie , et al. 2013b Evolution of mir‐92a underlies natural morphological variation in *Drosophila melanogaster* . Curr. Biol. 23:523–8. 23453955 10.1016/j.cub.2013.02.018 PMC3605577

[evl3115-bib-0005] Arnoult, L. , K. F. Y. Su , D. Manoel , C. Minervino , J. Magriña , N. Gompel , and B. Prud'homme 2013 Emergence and diversification of fly pigmentation through evolution of a gene regulatory module. Science 339:1423–6. 23520110 10.1126/science.1233749

[evl3115-bib-0006] Atallah, J. , L. Teixeira , R. Salazar , G. Zaragoza , and A. Kopp 2014a The making of a pest: the evolution of a fruit‐penetrating ovipositor in Drosophila suzukii and related species. Proc. R. Soc. Lond. B Biol. Sci. 281:20132840. 10.1098/rspb.2013.2840 PMC3953835 24573846

[evl3115-bib-0007] Atallah, J. , G. Vurens , S. Mavong , A. Mutti , D. Hoang , and A. Kopp 2014b Sex‐specific repression of dachshund is required for *Drosophila* sex comb development. Dev. Biol. 386:440–7. 24361261 10.1016/j.ydbio.2013.12.017

[evl3115-bib-0008] Bächli, G. , C. R. Vilela , S. Andersson Escher , and A. Saura 2004 The Drosophilidae (Diptera) of Fennoscandia and Denmark. Brill, Leiden, the Netherlands.

[evl3115-bib-0009] Blount, Z. D. , R. E. Lenski , and J. B. Losos 2018 Contingency and determinism in evolution: replaying life's tape. Science 362:eaam5979. 30409860 10.1126/science.aam5979

[evl3115-bib-0010] Bryant, H. N. 1995 Why autapomorphies should be removed: a reply to yeates. Cladistics 11:381–4. 10.1111/j.1096-0031.1995.tb00097.x 34920648

[evl3115-bib-0011] Charrad, M. , N. Ghazzali , V. Boiteau , and A. Niknafs 2014 NbClust: an R package for determining the relevant number of clusters in a data set. J. Stat. Softw. 61:1–36.

[evl3115-bib-0012] Civetta, A. , and R. S. Singh 1998 Sex and speciation: genetic architecture and evolutionary potential of sexual versus nonsexual traits in the sibling species of the *Drosophila melanogaster* complex. Evolution 52:1080–92. 28565206 10.1111/j.1558-5646.1998.tb01835.x

[evl3115-bib-0013] Conrad, J. L. 2008 Phylogeny and systematics of squamata (reptilia) based on morphology. Bull. Am. Mus. Nat. Hist. 10:1–183.

[evl3115-bib-0014] Conway Morris, S. 2008 The deep structure of biology: is convergence sufficiently ubiquitous to give a directional signal? Templeton Foundation Press, West Conshohocken, PA.

[evl3115-bib-0015] Cruickshank, T. , and M. J. Wade 2008 Microevolutionary support for a developmental hourglass: gene expression patterns shape sequence variation and divergence in *Drosophila* . Evol. Dev. 10:583–90. 18803776 10.1111/j.1525-142X.2008.00273.x

[evl3115-bib-0016] Darwin, C. 1859 On the origin of species by means of natural selection, or, the preservation of favoured races in the struggle for life. J. Murray, London. PMC5184128 30164232

[evl3115-bib-0017] Edgar, R. C. 2004 MUSCLE: multiple sequence alignment with high accuracy and high throughput. Nucleic Acids Res. 32:1792–7. 15034147 10.1093/nar/gkh340 PMC390337

[evl3115-bib-0018] Farris, J. S. 1989 The retention index and the rescaled consistency index. Cladistics 5:417–9. 10.1111/j.1096-0031.1989.tb00573.x 34933481

[evl3115-bib-0019] Frankel, N. , S. Wang , and D. L. Stern 2012 Conserved regulatory architecture underlies parallel genetic changes and convergent phenotypic evolution. Proc. Natl. Acad. Sci. USA 109:20975–9. 23197832 10.1073/pnas.1207715109 PMC3529038

[evl3115-bib-0020] Gauthier, J. A. , M. Kearney , J. A. Maisano , O. Rieppel , and A. D. B. Behlke 2012 Assembling the squamate tree of life: perspectives from the phenotype and the fossil record. Bull. Peabody Mus. Nat. Hist. 53:3–309.

[evl3115-bib-0021] Glassford, W. J. , W. C. Johnson , N. R. Dall , S. J. Smith , Y. Liu , W. Boll , M. Noll , et al. 2015 Co‐option of an ancestral hox‐regulated network underlies a recently evolved morphological novelty. Dev. Cell. 34:520–31. 26343453 10.1016/j.devcel.2015.08.005 PMC4573913

[evl3115-bib-0022] Godefroit, P. , A. Cau , H. Dong‐Yu , F. Escuillié , W. Wenhao , and G. Dyke 2013 A Jurassic avialan dinosaur from China resolves the early phylogenetic history of birds. Nature 498:359–62. 23719374 10.1038/nature12168

[evl3115-bib-0023] Green, D. A. , and C. G. Extavour 2012 Convergent evolution of a reproductive trait through distinct developmental mechanisms in *Drosophila* . Dev. Biol. 372:120–30. 23022298 10.1016/j.ydbio.2012.09.014

[evl3115-bib-0024] Grimaldi, D. A. 1990 A phylogenetic, revised classification of genera in the Drosophilidae (Diptera). Bull. Am. Mus. Nat. Hist. 197:1–139.

[evl3115-bib-0025] Grover, S. , M. E. Williams , R. Kaiser , J. T. Hughes , L. Gresham , M. Rebeiz , and T. M. Williams 2018 Augmentation of a wound response element accompanies the origin of a Hox‐regulated *Drosophila* abdominal pigmentation trait. Dev. Biol. 441:159–75. 29981311 10.1016/j.ydbio.2018.07.001 PMC6075670

[evl3115-bib-0026] Haerty, W. , S. Jagadeeshan , R. J. Kulathinal , A. Wong , K. R. Ram , L. K. Sirot , L. Levesque , et al. 2007 Evolution in the fast lane: rapidly evolving sex‐related genes in *Drosophila* . Genetics 177:1321–35. 18039869 10.1534/genetics.107.078865 PMC2147986

[evl3115-bib-0027] Hu, Y.‐G. , and M. J. Toda 2001 Polyphyly of Lordiphosa and its relationships in Drosophilinae (Diptera: Drosophilidae). Syst. Entomol. 26:15–31.

[evl3115-bib-0028] Irie, N. , and S. Kuratani 2014 The developmental hourglass model: a predictor of the basic body plan? Development 141:4649–55. 25468934 10.1242/dev.107318

[evl3115-bib-0029] Jeong, S. , M. Rebeiz , P. Andolfatto , T. Werner , J. True , and S. B. Carroll 2008 The evolution of gene regulation underlies a morphological difference between two *Drosophila* sister species. Cell 132:783–93. 18329365 10.1016/j.cell.2008.01.014

[evl3115-bib-0030] Jiménez‐Guri, E. , J. Huerta‐Cepas , L. Cozzuto , K. R. Wotton , H. Kang , H. Himmelbauer , G. Roma , et al. 2013 Comparative transcriptomics of early dipteran development. BMC Genomics 14:123. 23432914 10.1186/1471-2164-14-123 PMC3616871

[evl3115-bib-0031] Kagesawa, T. , Y. Nakamura , M. Nishikawa , Y. Akiyama , M. Kajiwara , and K. Matsuno 2008 Distinct activation patterns of EGF receptor signaling in the homoplastic evolution of eggshell morphology in genus *Drosophila* . Mech. Dev. 125:1020–32. 18762251 10.1016/j.mod.2008.08.001

[evl3115-bib-0032] Kalinka, A. T. , K. M. Varga , D. T. Gerrard , S. Preibisch , D. L. Corcoran , J. Jarrells , U. Ohler , et al. 2010 Gene expression divergence recapitulates the developmental hourglass model. Nature 468:811–4. 21150996 10.1038/nature09634

[evl3115-bib-0033] Kamimura, Y. 2007 Twin intromittent organs of *Drosophila* for traumatic insemination. Biol. Lett. 3:401–4. 17519186 10.1098/rsbl.2007.0192 PMC2391172

[evl3115-bib-0034] Kluge, A. G. , and J. S. Farris 1969 Quantitative phyletics and the evolution of anurans. Syst. Biol. 18:1–32.

[evl3115-bib-0035] Kumar, S. , G. Stecher , and K. Tamura 2016 MEGA7: molecular evolutionary genetics analysis version 7.0 for bigger datasets. Mol. Biol. Evol. 33:1870–4. 27004904 10.1093/molbev/msw054 PMC8210823

[evl3115-bib-0036] Laing, A. M. , S. Doyle , M. E. L. Gold , S. J. Nesbitt , M. A. O'Leary , A. H. Turner , E. W. Wilberg , et al. 2018 Giant taxon‐character matrices: the future of morphological systematics. Cladistics 34:333–5. 10.1111/cla.12197 34645074

[evl3115-bib-0037] Lambkin, C. L. , B. J. Sinclair , T. Pape , G. W. Courtney , J. H. Skevington , R. Meier , D. K. Yeates , et al. 2013 The phylogenetic relationships among infraorders and superfamilies of Diptera based on morphological evidence. Syst. Entomol. 38:164–79.

[evl3115-bib-0038] Lewis, P. O. 2001 A likelihood approach to estimating phylogeny from discrete morphological character data. Syst. Biol. 50:913–25. 12116640 10.1080/106351501753462876

[evl3115-bib-0039] Livezey, B. C. , and R. L. Zusi 2007 Higher‐order phylogeny of modern birds (Theropoda, Aves: Neornithes) based on comparative anatomy. II. Analysis and discussion. Zool. J. Linn. Soc. 149:1–95. 18784798 10.1111/j.1096-3642.2006.00293.x PMC2517308

[evl3115-bib-0040] Marcellini, S. , and P. Simpson 2006 Two or four bristles: functional evolution of an enhancer of scute in Drosophilidae. PLoS Biol. 4:e386. 17105353 10.1371/journal.pbio.0040386 PMC1635746

[evl3115-bib-0041] Martin, A. , and V. Orgogozo 2013 The Loci of repeated evolution: a catalog of genetic hotspots of phenotypic variation. Evol. Int. J. Org. Evol. 67:1235–50. 10.1111/evo.12081 23617905

[evl3115-bib-0042] Massey, J. H. , and P. J. Wittkopp 2016 Chapter two—the genetic basis of pigmentation differences within and between *Drosophila* species Pp. 27–61 *in* Orgogozo V., ed. Current topics in developmental biology. Academic Press, Cambridge, MA. 10.1016/bs.ctdb.2016.03.004 PMC5002358 27282023

[evl3115-bib-0043] McGhee, G. R. 2011 Convergent evolution: Limited forms most beautiful. MIT Press, Cambridge, MA, pp 335.

[evl3115-bib-0044] Nagy, O. , I. Nuez , R. Savisaar , A. E. Peluffo , A. Yassin , M. Lang , D. L. Stern , et al. 2018 Correlated evolution of two sensory organs via a single cis‐regulatory nucleotide change. Curr. Biol. 28:3450–3467. 30344115 10.1016/j.cub.2018.08.047 PMC7385753

[evl3115-bib-0045] Niepielko, M. G. , and N. Yakoby 2014 Evolutionary changes in TGFα distribution underlie morphological diversity in eggshells from *Drosophila* species. Development. 141:4710–5. 25468939 10.1242/dev.111898 PMC4299280

[evl3115-bib-0046] O'Grady, P. M. , and R. DeSalle 2018 Phylogeny of the genus *Drosophila* . Genetics. 209:1–25. 29716983 10.1534/genetics.117.300583 PMC5937177

[evl3115-bib-0047] Okada, T. 1968 Systematic study of the early stages of Drosophilidae. Bunka Zugeisha, Tokyo, Japan.

[evl3115-bib-0048] O'Leary, M. A. , J. I. Bloch , J. J. Flynn , T. J. Gaudin , A. Giallombardo , N. P. Giannini , S. L. Goldberg , et al. 2013 The placental mammal ancestor and the post–K‐Pg radiation of placentals. Science 339:662–667. 23393258 10.1126/science.1229237

[evl3115-bib-0049] Orgogozo, V. , K. W. Broman , and D. L. Stern 2006 High‐resolution quantitative trait locus mapping reveals sign epistasis controlling ovariole number between two *Drosophila* species. Genetics 173:197–205. 16489225 10.1534/genetics.105.054098 PMC1461429

[evl3115-bib-0050] Orgogozo, V. , N. M. Muro , and D. L. Stern 2007 Variation in fiber number of a male‐specific muscle between *Drosophila* species: a genetic and developmental analysis. Evol. Dev. 9:368–77. 17651361 10.1111/j.1525-142X.2007.00174.x

[evl3115-bib-0051] Osterfield, M. , T. Schüpbach , E. Wieschaus , and S. Y. Shvartsman 2015 Diversity of epithelial morphogenesis during eggshell formation in drosophilids. Development. 142:1971–7. 25953345 10.1242/dev.119404 PMC4460739

[evl3115-bib-0052] Parsch, J. , and H. Ellegren 2013 The evolutionary causes and consequences of sex‐biased gene expression. Nat. Rev. Genet. 14:83–7. 23329110 10.1038/nrg3376

[evl3115-bib-0053] Patterson, J. T. , and W. S. Stone 1952 Evolution in the genus *Drosophila*. Macmillan New York.

[evl3115-bib-0054] Pearce, T. 2012 Convergence and parallelism in evolution: A Neo‐Gouldian account. Br. J. Philos. Sci. 63:429–48.

[evl3115-bib-0055] Peluffo, A. E. , I. Nuez , V. Debat , R. Savisaar , D. L. Stern , and V. Orgogozo 2015 A major locus controls a genital shape difference involved in reproductive isolation between *Drosophila yakuba* and *Drosophila santomea* . G3 5:2893–2901. 26511499 10.1534/g3.115.023481 PMC4683660

[evl3115-bib-0056] Powell, R. , and C. Mariscal 2015 Convergent evolution as natural experiment: the tape of life reconsidered. Interface Focus 5:20150040. 26640647 10.1098/rsfs.2015.0040 PMC4633857

[evl3115-bib-0057] Prud'homme, B. , N. Gompel , A. Rokas , V. A. Kassner , T. M. Williams , S.‐D. Yeh , J. R. True , et al. 2006 Repeated morphological evolution through *cis*‐regulatory changes in a pleiotropic gene. Nature 440:1050–3. 16625197 10.1038/nature04597

[evl3115-bib-0058] Rebeiz, M. , J. E. Pool , V. A. Kassner , C. F. Aquadro , and S. B. Carroll 2009 Stepwise modification of a modular enhancer underlies adaptation in a *Drosophila* population. Science 326:1663–7. 20019281 10.1126/science.1178357 PMC3363996

[evl3115-bib-0059] Rice, G. , O. Barmina , K. Hu , and A. Kopp 2018 Evolving doublesex expression correlates with the origin and diversification of male sexual ornaments in the Drosophila immigrans species group. Evol. Dev. 20:78–88. 29372584 10.1111/ede.12249 PMC6444933

[evl3115-bib-0060] Ronquist, F. , M. Teslenko , P. van der Mark , D. L. Ayres , A. Darling , S. Höhna , B. Larget , et al. 2012 MrBayes 3.2: efficient bayesian phylogenetic inference and model choice across a large model space. Syst. Biol. 61:539–42. 22357727 10.1093/sysbio/sys029 PMC3329765

[evl3115-bib-0061] Schep, A. N. , and B. Adryan 2013 A comparative analysis of transcription factor expression during metazoan embryonic development. PLoS One 8:e66826. 23799133 10.1371/journal.pone.0066826 PMC3682979

[evl3115-bib-0062] Signor, S. A. , Y. Liu , M. Rebeiz , and A. Kopp 2016 Genetic convergence in the evolution of male‐specific color patterns in Drosophila. Curr. Biol. 26:2423–33. 27546578 10.1016/j.cub.2016.07.034

[evl3115-bib-0063] Simões, T. R. , M. W. Caldwell , A. Palci , and R. L. Nydam 2017 Giant taxon‐character matrices: quality of character constructions remains critical regardless of size. Cladistics 33:198–219. 10.1111/cla.12163 34710972

[evl3115-bib-0064] Song, H. , and S. R. Bucheli 2010 Comparison of phylogenetic signal between male genitalia and non‐genital characters in insect systematics. Cladistics 26:23–35. 10.1111/j.1096-0031.2009.00273.x 34875749

[evl3115-bib-0065] Speed, M. P. , and K. Arbuckle 2017 Quantification provides a conceptual basis for convergent evolution. Biol. Rev. 92:815–29. 26932796 10.1111/brv.12257 PMC6849873

[evl3115-bib-0066] Stayton, C. T. 2015 What does convergent evolution mean? The interpretation of convergence and its implications in the search for limits to evolution. Interface Focus 5:20150039. 26640646 10.1098/rsfs.2015.0039 PMC4633856

[evl3115-bib-0067] Sturtevant, A. H. 1921 The North American species of Drosophila. Carnegie Inst. Wash. Publ. 301:1–150.

[evl3115-bib-0068] Swofford, D. L. 2002 PAUP*. Phylogenetic analysis using parsimony (*and other methods). Version 4. Sinauer Associates, Sunderland, MA.

[evl3115-bib-0069] Tanaka, K. , O. Barmina , and A. Kopp 2009 Distinct developmental mechanisms underlie the evolutionary diversification of Drosophila sex combs. Proc. Natl. Acad. Sci. USA 106:4764–4769. 19255422 10.1073/pnas.0807875106 PMC2660783

[evl3115-bib-0070] Tanaka, K. M. , C. Hopfen , M. R. Herbert , C. Schlötterer , D. L. Stern , J. P. Masly , A. P. McGregor , et al. 2015 Genetic Architecture and functional characterization of genes underlying the rapid diversification of male external genitalia between *Drosophila simulans* and *Drosophila mauritiana* . Genetics 200:357–369. 25783699 10.1534/genetics.114.174045 PMC4423377

[evl3115-bib-0071] Throckmorton, L. H. 1962 The problem of phylogeny in the genus Drosophila. Univ. Tex. Publ. 6205:207–343.

[evl3115-bib-0072] Vogt, L. , T. Bartolomaeus , and G. Giribet 2010 The linguistic problem of morphology: structure versus homology and the standardization of morphological data. Cladistics 26:301–325. 10.1111/j.1096-0031.2009.00286.x 34875783

[evl3115-bib-0073] Wake, D. B. , M. H. Wake , and C. D. Specht 2011 Homoplasy: From Detecting Pattern to Determining Process and Mechanism of Evolution. Science 331:1032–1035. 21350170 10.1126/science.1188545

[evl3115-bib-0074] Werner, T. , S. Koshikawa , T. M. Williams , and S. B. Carroll 2012 Generation of a novel wing colour pattern by the Wingless morphogen. Nature 464:1143–1148. 10.1038/nature08896 20376004

[evl3115-bib-0075] Wittkopp, P. J. , S. B. Carroll , and A. Kopp 2003a Evolution in black and white: genetic control of pigment patterns in Drosophila. Trends Genet. 19:495–504. 12957543 10.1016/S0168-9525(03)00194-X

[evl3115-bib-0076] Wittkopp, P. J. , B. L. Williams , J. E. Selegue , and S. B. Carroll 2003b Drosophila pigmentation evolution: divergent genotypes underlying convergent phenotypes. Proc. Natl. Acad. Sci. USA 100:1808–1813. 12574518 10.1073/pnas.0336368100 PMC149915

[evl3115-bib-0077] Xie, W. , P. O. Lewis , Y. Fan , L. Kuo , and M.‐H. Chen 2011 Improving marginal likelihood estimation for bayesian phylogenetic model selection. Syst. Biol. 60:150–160. 21187451 10.1093/sysbio/syq085 PMC3038348

[evl3115-bib-0078] Yassin, A. 2013 Phylogenetic classification of the Drosophilidae Rondani (Diptera): the role of morphology in the postgenomic era. Syst. Entomol. 38:349–364.

[evl3115-bib-0079] Yassin, A. 2016 Unresolved questions in genitalia coevolution: bridging taxonomy, speciation, and developmental genetics. Org. Divers. Evol. 16:681–688.

[evl3115-bib-0080] Yassin, A. , H. Bastide , H. Chung , M. Veuille , J. R. David , and J. E. Pool 2016a Ancient balancing selection at tan underlies female colour dimorphism in Drosophila erecta. Nat. Commun. 7:10400. 26778363 10.1038/ncomms10400 PMC4735637

[evl3115-bib-0081] Yassin, A. , E. K. Delaney , A. J. Reddiex , T. D. Seher , H. Bastide , N. C. Appleton , J. B. Lack , et al. 2016b The pdm3 locus is a hotspot for recurrent evolution of female‐limited color dimorphism in Drosophila. Curr. Biol. 26:2412–2422. 27546577 10.1016/j.cub.2016.07.016 PMC5450831

[evl3115-bib-0082] Yassin, A. , E. K. Lienau , A. Narechania , and R. DeSalle 2010 Catching the phylogenic history through the ontogenic hourglass: a phylogenomic analysis of Drosophila body segmentation genes. Evol. Dev. 12:288–295. 20565539 10.1111/j.1525-142X.2010.00414.x

[evl3115-bib-0083] Yassin, A. , and V. Orgogozo 2013 Coevolution between male and female genitalia in the Drosophila melanogaster species subgroup. PLoS One 8:e57158. 23451172 10.1371/journal.pone.0057158 PMC3581563

[evl3115-bib-0084] Zou, Z. , and J. Zhang 2016 Morphological and molecular convergences in mammalian phylogenetics. Nat. Commun. 7:12758. 27585543 10.1038/ncomms12758 PMC5025827

