# Supplementary material for: Quantifying the extent of morphological homoplasy: A phylogenetic analysis of 490 characters in Drosophila
Source: Evol Lett. 2019 Apr 22;3(3):286–98. doi: 10.1002/evl3.115 (PMC6546384; doi:10.1002/evl3.115)
Supplement: Supplementary file 1 — Supporting Information [file EVL3-3-286-s001.pdf]

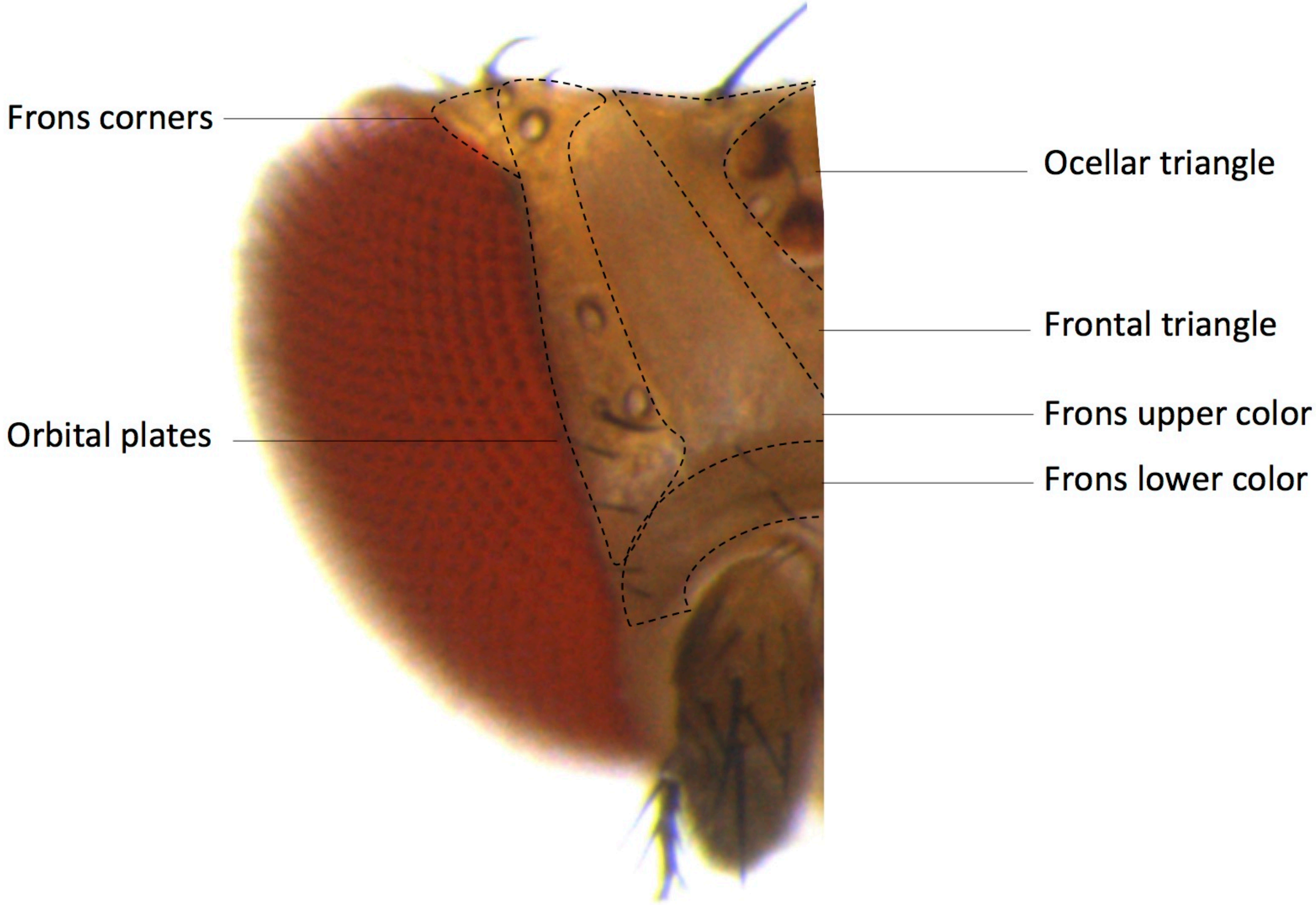
Frons corners
Orbital plates
Ocellar triangle
Frontal triangle
Frons upper color
Frons lower color

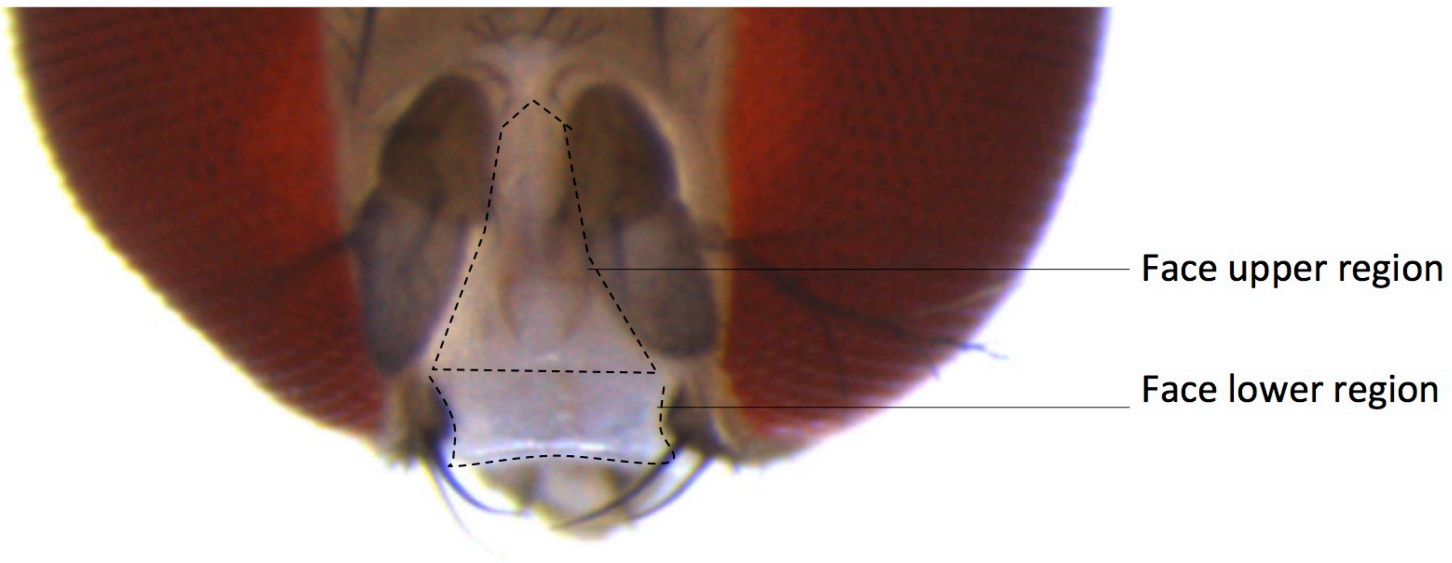
Face upper region
Face lower region

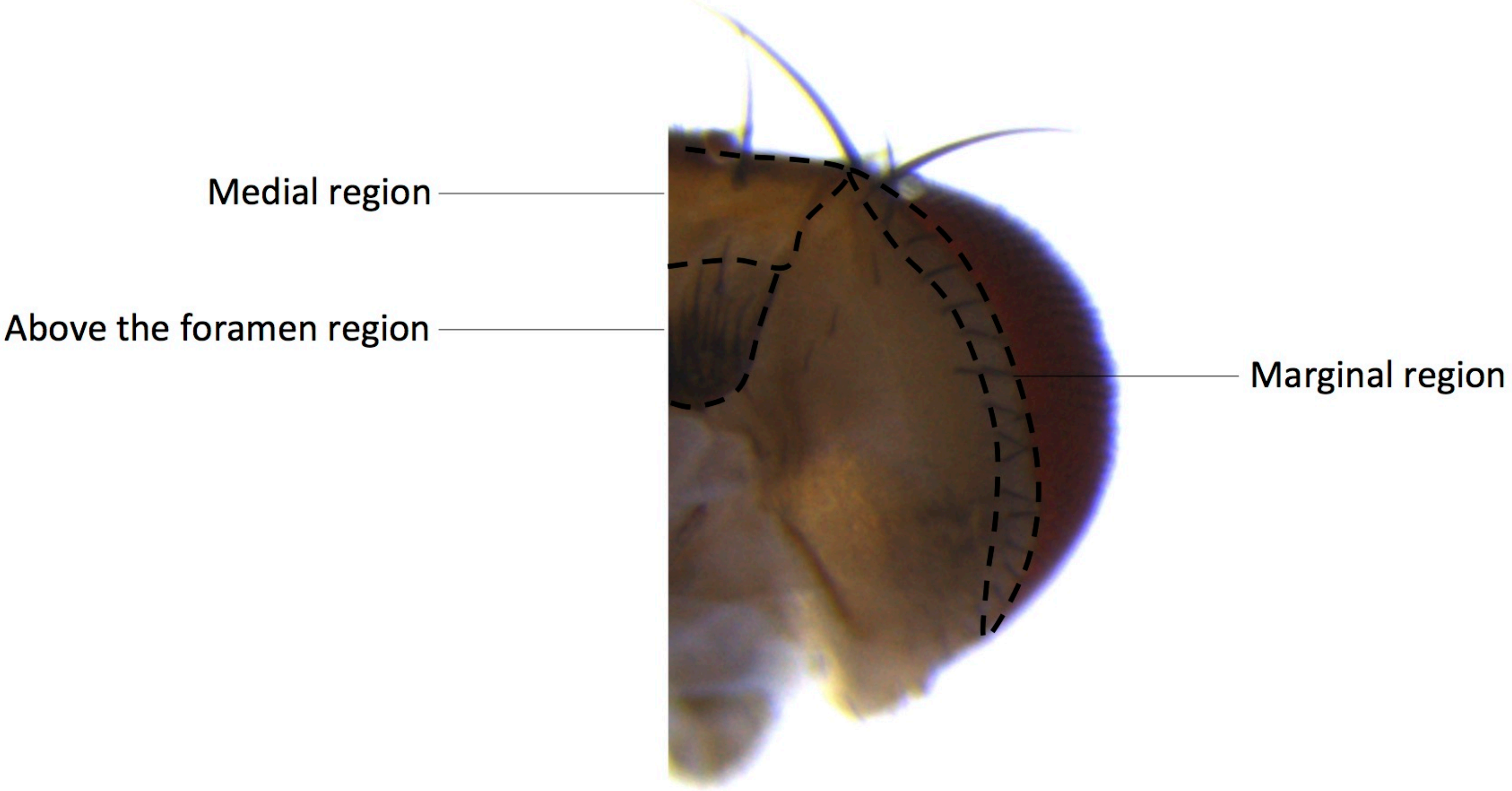
Medial region
Above the foramen region
Marginal region

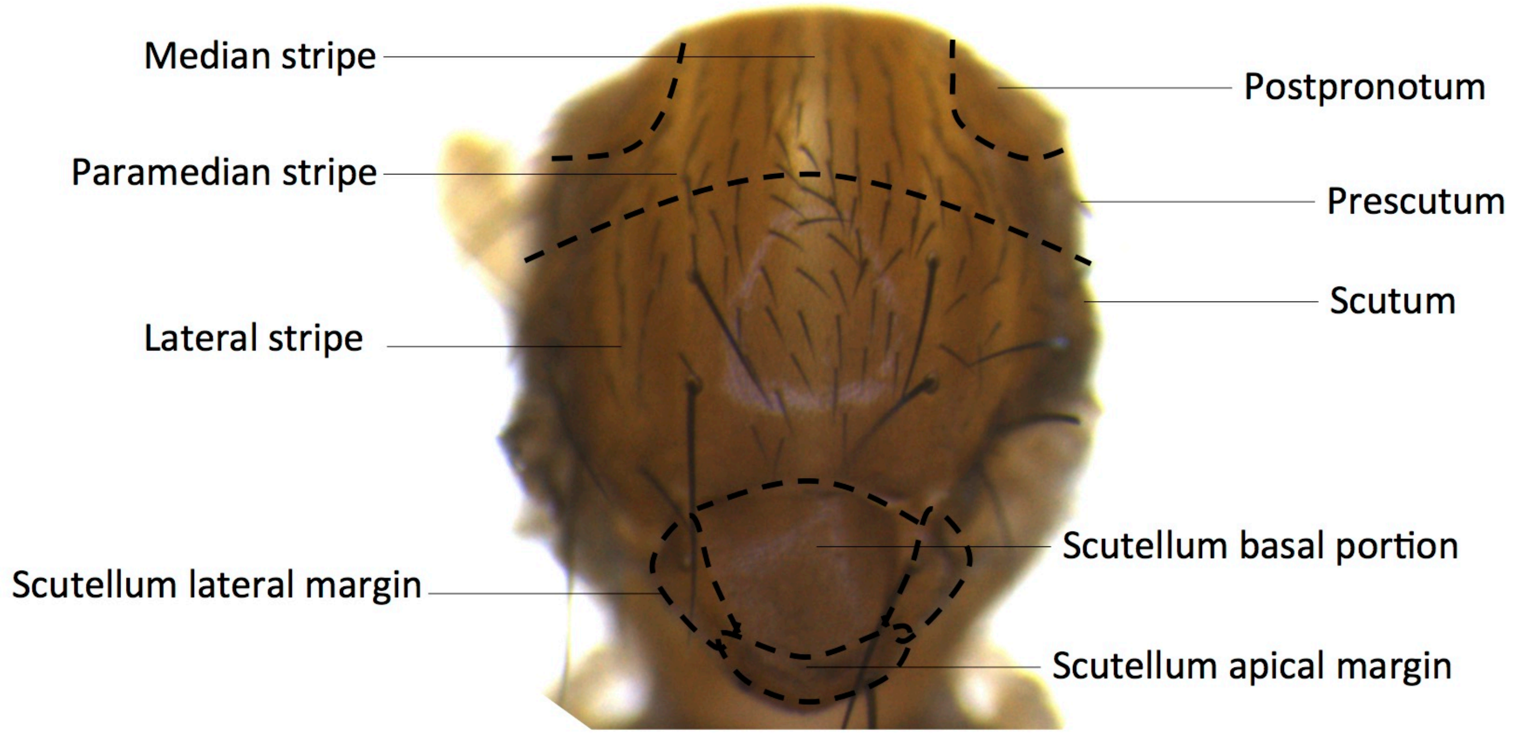
Median stripe
Paramedian stripe
Lateral stripe
Scutellum lateral margin
Postpronotum
Prescutum
Scutum
Scutellum basal portion
Scutellum apical margin

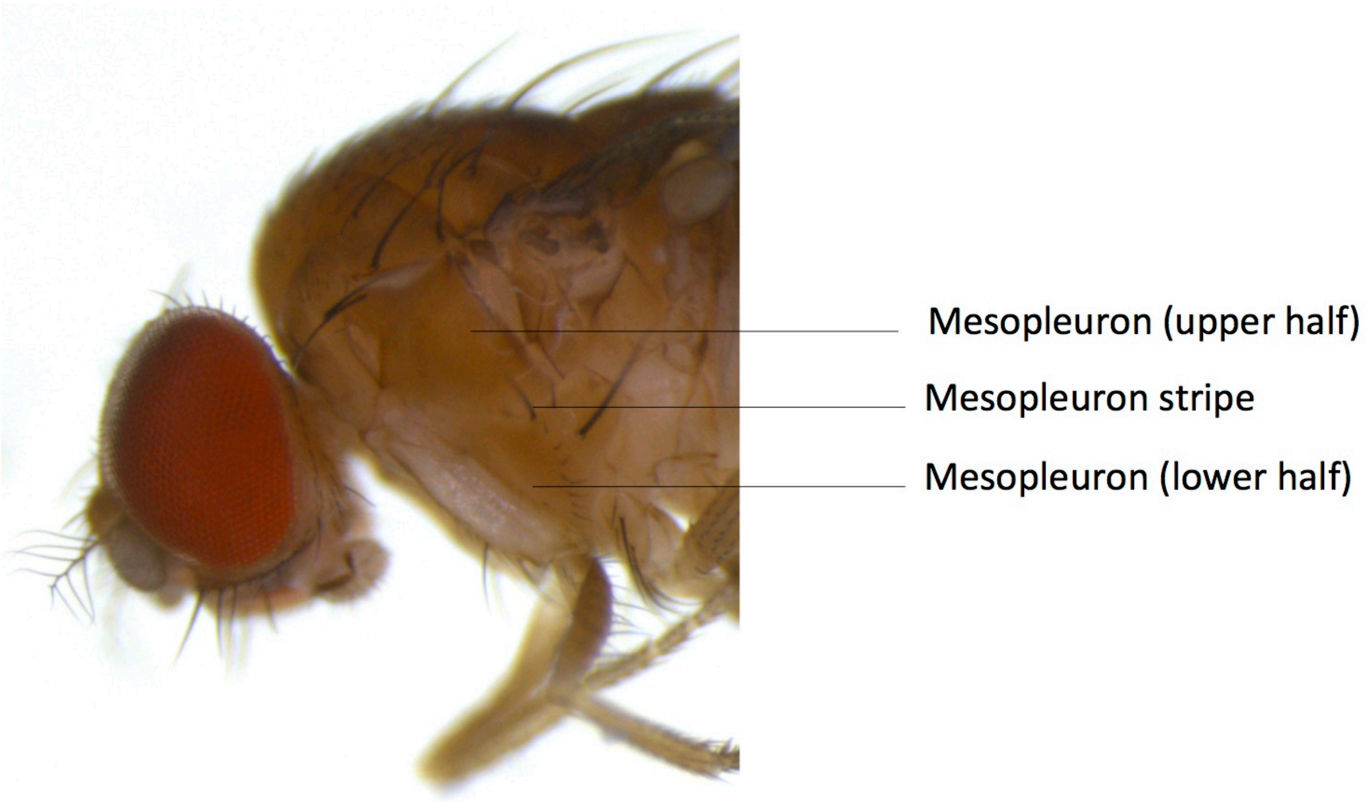
Mesopleuron (upper half)
Mesopleuron stripe
Mesopleuron (lower half)

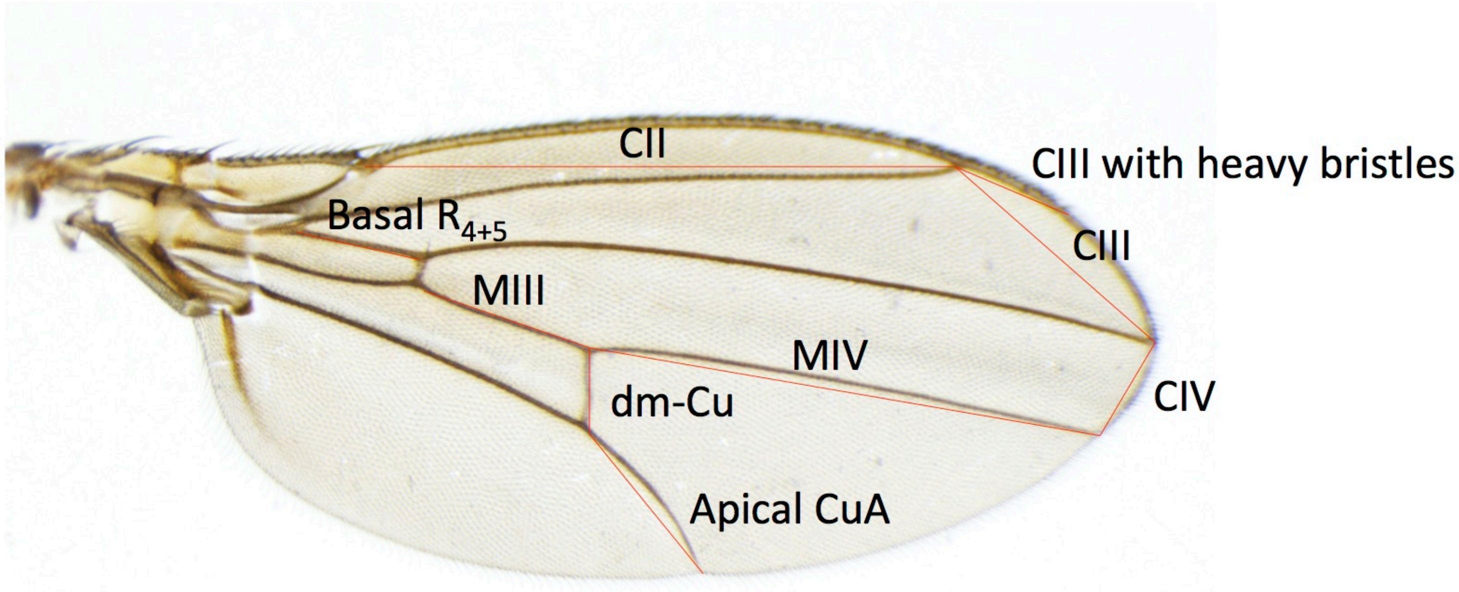
CII
CIII with heavy bristles
Basal R4+5
CIII
MIII
MIV
CIV
dm-Cu
Apical CuA

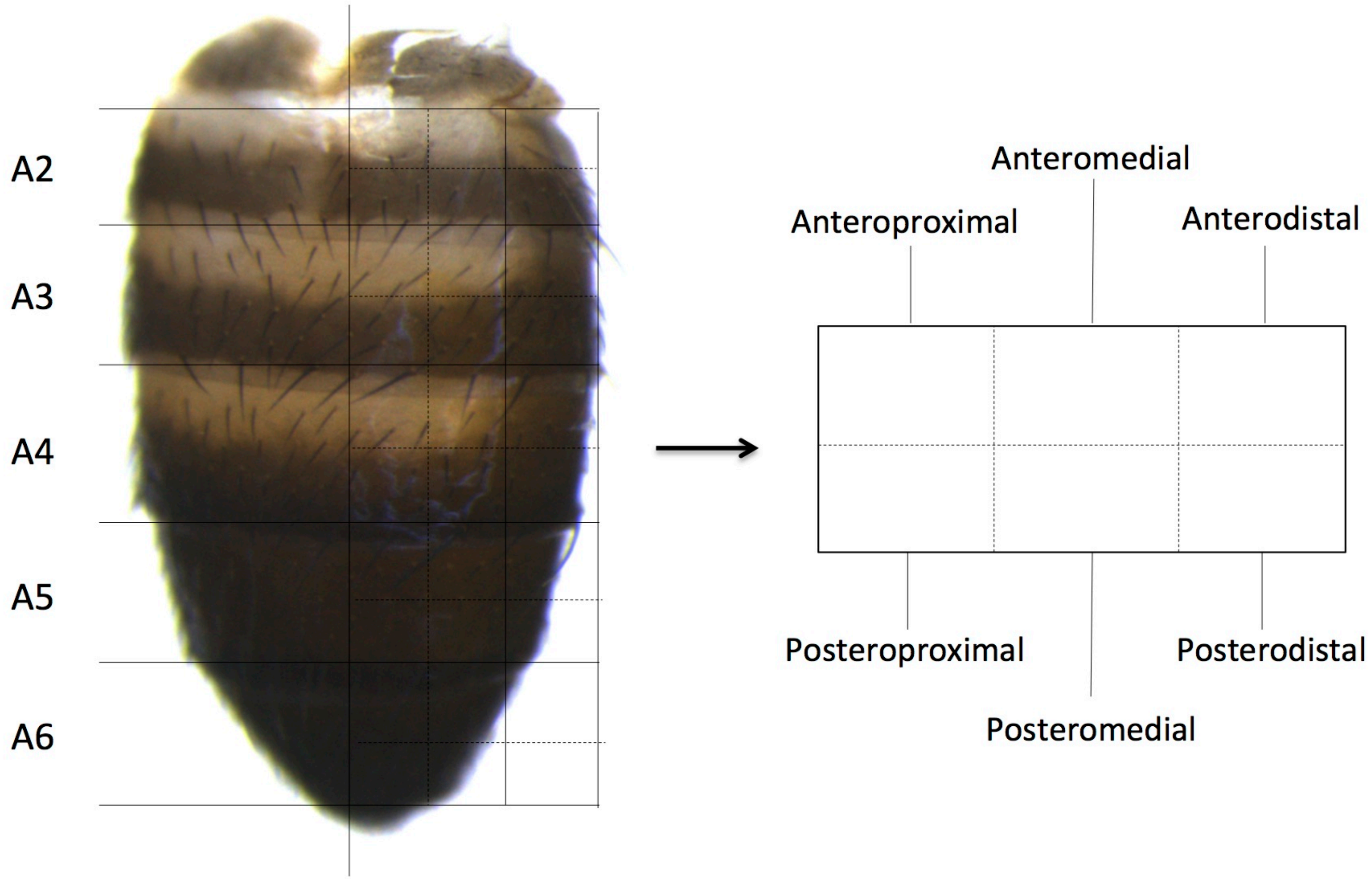
A2
A3
A4
A5
A6
Anteroproximal
Anteromedial
Anterodistal
Posteroproximal
Posteromedial
Posterodistal
