# Supplementary material for: Quantifying the extent of morphological homoplasy: A phylogenetic analysis of 490 characters in Drosophila
Source: Evol Lett. 2019 Apr 22;3(3):286–98. doi: 10.1002/evl3.115 (PMC6546384; doi:10.1002/evl3.115)
Supplement: Supplementary file 6 — Supporting Information [file EVL3-3-286-s006.pdf]

Character definitions and character state codes

<u>Egg</u>

1. Color: 0=white.
2. Length, distance from the anterior to the posterior ends in mm: 0=0.4 to 0.45; 1=0.5 to 0.57; 2=0.6 to 0.8.
3. Shape, ratio of egg length to egg width at mid-length: 0=1.71 to 1.71; 1=2.16 to 2.77; 2=2.82 to 3.44.
4. Dorsal shape, angle between the dorsal margin of the egg posterior to the opercullar collar and a line connecting the opercullar collar to the anterior end: 0=124 to 131; 1=138 to 146; 2=147 to 160.
5. Dorsal longitudinal structures: 0=absent; 1=ridges; 2=vela.
6. Micropyle fringe: 0=absent; 1=present.
7. Filaments number: 0=0 to 0; 1=2 to 2; 2=3 to 4; 3=3 to 6; 4=6 to 12.
8. Filaments shape: 0=dilated; 1=filiform.
9. Filaments length, ratio in length of the longest filament and the egg: 0=0.12 to 0.4; 1=0.5 to 0.7; 2=0.75 to 1.2.
10. Filaments differentiation, ratio between the length of the anterior filament to the posterior ones in species with 3 or 4 filaments: 0=equal; 1=longer; 2=much shorter; 3=shorter; 4=slightly shorter; 5=somewhat shorter.
11. Surface sculpture, number of imprints of the follicle cells per longitudinal row: 0=15 to 20; 1=22 to 30; 2=45 to 45.
12. Surface between vela: 0=not striated; 1=striated.

<u>Larva (1st instar)</u>

13. Ventral abdominal hooklets color: 0=black; 1=grey; 2=white.
14. Posterior spiracles pigmentation: 0=hyaline; 1=pigmented.
15. Posterior spiracles color: 0=black; 1=white; 2=yellow.
16. Cephalopharyngeal skeleton length in mm: 0=0.13 to 0.18; 1=0.2 to 0.25; 2=0.5 to 0.5.
17. Mouth hook teeth: 0=0 to 1; 1=2 to 3; 2=4 to 5.
18. Mouth hook teeth size: 0=Exceedingly large; 1=not large.
19. Mouth hook teeth type (as defined by Okada 1968): 0=with one ventral tooth; 1=with one ventral tooth/with several acute prominent teeth; 2=with several acute prominent teeth; 3=without ventral tooth; 4=without ventral tooth/with one ventral tooth; 5=without ventral tooth/with several acute prominent teeth.
20. Ventral process size: 0=slender; 1=thick and triangular.
21. Ventral process shape, angle between the line connecting the tip of the ventral process to the middle of the ventral margin of the mouth hook and the line connecting the tip of the ventral process to the middle of the posterior margin: 0=13 to 17; 1=20 to 28; 2=31 to 40; 3=46 to 56.
22. Ventral process position, ratio between the length of the anterior margin of the ventral process and the length of the ventral margin of the anterior process: 0=0.5; 1=0.6; 2=0.7.
23. Vertical bridge/dorsal wing, ratio between the length of the vertical bridge and the length of the dorsal arm of the pharyngeal sclerite: 0=0.1 to 0.16; 1=0.2 to 0.2; 2=0.25 to 0.33.

24. Vertical bridge evagination, angle between the dorsal margin of dorsal wing and the line connecting the anterior tip of the dorsal wing and the basis of latticed process on the mid-anterior margin of the vertical bridge: 0=22 to 40; 1=42 to 60.
25. Vertical bridge anterior shape: 0=concacve; 1=convex.

Larva (2nd instar)
26. Ventral abdominal hooklets color: 0=black; 1=grey; 2=white.
27. Posterior spiracles color: 0=black; 1=grey; 2=white; 3=yellow.
28. Cephalopharyngeal skeleton length in mm: 0=0.23 to 0.3; 1=0.32 to 0.5; 2=0.7 to 0.7.
29. Mouth hook teeth: 0=0 to 3; 1=4 to 6; 2=8 to 15.
30. Mouth hook teeth size: 0=Exceedingly large; 1=not large.
31. Mouth hook teeth type (cf. 19): 0=with numerous minute teeth; 1=with one ventral tooth; 2=with one ventral tooth/with several acute prominent teeth; 3=with several acute prominent teeth.
32. Ventral process position (cf. 22): 0=0.5; 1=0.6; 2=0.7.
33. Ventral process shape (cf. 21): 0=28 to 43; 1=45 to 60; 2=62 to 80.
34. Epipharyngeal sclerite shape: 0=pentagonal; 1=quadrate.
35. Epipharyngeal sclerite anterior shape: 0=not pointed; 1=pointed.
36. Hypopharyngeal sclerite width: 0=broad; 1=slender.
37. Hypopharyngeal sclerite shape: 0=fusiform or elliptical; 1=triangular.
38. Hypopharyngeal sclerite anterior shape: 0=narrowing; 1=not pointed or narrowing; 2=pointed.
39. Hypopharyngeal sclerite medial shape: 0=constricted; 1=not constricted.
40. Latticed process size: 0=broad; 1=narrow.
41. Latticed process anterior shape: 0=not pointed; 1=pointed.
42. Latticed process medial shape: 0=constricted; 1=not constricted; 2=somewhat constricted.
43. Latticed process/dorsal wing connection: 0=fused; 1=separated.
44. Latticed process foramens number: 0=0 to 0; 1=3 to 6; 2=7 to 8; 3=9 to 10.
45. Latticed process foramens size: 0=large or elongate; 1=moderate; 2=small.
46. Vertical bridge/dorsal wing, ratio in length: 0=0.12 to 0.2; 1=0.25 to 0.33; 2=0.66 to 0.66.
47. Vertical bridge evagination, angle between the dorsal margin of dorsal wing and the line connecting the anterior tip of the dorsal wing and the basis of latticed process on the mid-anterior margin of the vertical bridge: 0=31 to 58; 1=62 to 84; 2=88 to 109.
48. Vertical bridge evagination shape: 0=large and exceedingly convexed; 1=weakly concaved.

Larva (3rd instar)
49. Ventral abdominal hooklets color: 0=black; 1=grey; 2=white.
50. Posterior spiracles color: 0=black; 1=grey; 2=tan; 3=white; 4=yellow.
51. Caphalopharyngeal skeleton length in mm: 0=0.17 to 0.17; 1=0.37 to 0.57; 2=0.6 to 0.9.
52. Mouth hook teeth: 0=0 to 1; 1=17 to 20; 2=3 to 6; 3=9 to 15.
53. Mouth hook teeth type (cf. 19): 0=with a few proximal large teeth and numerous distal minute teeth; 1=with a proximal large tooth and numerous minute teeth/with numerous minute teeth; 2=with numerous minute teeth; 3=with several acute prominent teeth; 4=without ventral tooth.

54. Ventral process position (cf. 22): 0=0.3; 1=0.5; 2=0.6.
55. Ventral process shape (cf. 21): 0=36 to 50; 1=52 to 60; 2=63 to 76; 3=88 to 95.
56. Epipharyngeal sclerite shape: 0=oval; 1=pentagonal; 2=quadrate.
57. Epipharyngeal sclerite anterior shape: 0=pointed; 1=rounded.
58. Hypopharyngeal sclerite width: 0=broad; 1=slender.
59. Hypopharyngeal sclerite shape: 0=fusiform; 1=rhombic or elliptical; 2=triangular.
60. Hypopharyngeal sclerite anterior shape: 0=narrowing to pointed; 1=not pointed.
61. Hypopharyngeal sclerite medial shape: 0=constricted; 1=not constricted.
62. Hypopharyngeal sclerite posterior margin: 0=not tapering; 1=tapering.
63. Hypopharyngeal sclerite sclerotization: 0=thick; 1=thin.
64. Latticed process size: 0=broad; 1=narrow.
65. Latticed process anterior shape: 0=not pointed; 1=pointed.
66. Latticed process/dorsal wing connection: 0=fused; 1=separated.
67. Latticed process foramens number: 0=10 to 12; 1=3 to 9.
68. Latticed process foramens size: 0=large; 1=moderate; 2=small.
69. Vertical bridge/dorsal wing, ratio in length: 0=0.11 to 0.166; 1=0.2 to 0.25; 2=0.33 to 0.33.
70. Vertical bridge evagination, angle between the dorsal margin of dorsal wing and the line connecting the anterior tip of the dorsal wing and the basis of latticed process on the mid-anterior margin of the vertical bridge: 0=29 to 51; 1=56 to 77; 2=82 to 107.
71. Vertical bridge anterior shape: 0=with large conical evagination; 1=without conical evagination.
72. Vertical bridge evagination protrusion: 0=from the dorsoanterior margin of ventral wing; 1=not from the dorsoanterior margin of ventral wing.
73. Hypostomal sclerite/latticed process connection: 0=fused to the latticed process; 1=separated.
74. Body tubercles: 0=absent; 1=present.

<u>Puparium</u>
75. Color: 0=black; 1=grey; 2=tan; 3=white; 4=yellow; 5=yellowish.
76. Length, distance from the anterior margin (exclusive of the anterior spiracles) to the tip of the posterior spiracles in mm: 0=2 to 2.5; 1=2.7 to 3; 2=3.2 to 4; 3=4.2 to 5.
77. Shape, ratio of puparial length to puparial width at mid-length: 0=2.21 to 2.57; 1=2.65 to 3.25; 2=3.59 to 3.76.
78. Lateral margin shape: 0=not waving; 1=waving.
79. Hooklets type (as defined by Okada 1968): 0=divided into anterior broad linear transverse rows and posterior medially convergent posterior rows; 1=divided into medially divergent anterior rows and medially convergent posterior rows; 2=in a narrow linear transverse rows; 3=in a narrow medioanteriorly convened rows; 4=in medially convergent rows; 5=scattering evenly on the entire tergite; 6=without prominent hooklets.
80. Dorsal shape, angle between the dorsal margin of the puparium posterior to the opercullar collar and a line connecting the opercullar collar to the anterior margin: 0=121 to 136; 1=140 to 178.
81. Dorsal abdominal hooklets number: 0=10 to 15; 1=3 to 6; 2=7 to 9.
82. Ventral abdominal hooklets number: 0=10 to 11; 1=4 to 4; 2=7 to 7; 3=8 to 9.
83. Anterior spiracle stalk color: 0=black; 1=grey; 2=tan; 3=white; 4=yellow.
84. Anterior spiracle stalk length/width ratio: 0=0.2 to 1; 1=2 to 5; 2=25 to 25; 3=8 to 10.

85. Anterior spiracle lip projection: 0=absent; 1=present.
86. Anterior spiracle branches color: 0=black; 1=white; 2=yellow.
87. Anterior spiracles branches distal shape (as defined by Okada 1968): 0=conical; 1=pointed; 2=hooked; 3=clubbed.
88. Anterior spiracles branches dorsal shape, angle between the axis of the tip of the branch and the main branch axis: 0=118 to 180; 1=26 to 86.
89. Anterior spiracles branches number: 0=10 to 14; 1=16 to 28; 2=5 to 9.
[The branches of the anterior spiracles are divided into four types: basals, centrals pseudocentrals, marginal and pseudobasals.]
90. Basals length relative to other branches: 0=equal; 1=long; 2=minute; 3=short.
91. Basals shape: 0=not recurved; 1=recurved; 2=somewhat recurved.
92. Gap between basals: 0=intermediate; 1=narrow; 2=wide.
93. Centrals number: 0=0 to 0; 1=1 to 3; 2=10 to 10; 3=4 to 6.
94. Centrals length relative to other branches: 0=long; 1=short.
95. Pseudocentrals number: 0=0 to 0; 1=2 to 2.
96. Pseudocentrals length relative to other branches: 0=equal; 1=long; 2=short.
97. Marginals number: 0=1 to 5; 1=12 to 18; 2=6 to 10.
98. Marginals length relative to other branches: 0=gradually long; 1=long; 2=short.
99. Pseudobasals number: 0=0 to 0; 1=2 to 2; 2=4 to 4.
100. Pseudobasals length relative to other branches and other pseudobasals: 0=2 short and 2 long; 1=long.
101. Anterior spiracles branches disposition: 0=in a whorl; 1=in an elongate whorl; 2=not in a whorl.
102. Anterior spiracles branches texture: 0=smooth; 1=wrinkled.
103. Anterior spiracles longest branch/stalk ratio: 0=0.14 to 0.6; 1=0.8 to 1.5; 2=1.7 to 3.
104. Horn index, ratio in length between the puparial length (without the anterior spiracles) and the length of the anterior spiracles: 0=1.7 to 8; 1=20 to 35; 2=45 to 50; 3=9 to 15.
105. Posterior spiracles shape: 0=conical or pointed; 1=rod.
106. posterior spiracles color: 0=black; 1=grey; 2=tan; 3=white; 4=yellow.
107. posterior spiracles divergence: 0=closed; 1=closely divergent; 2=divergent.
108. Posterior spiracles connection: 0=attached at bases; 1=separated at bases.
109. Posterior spiracles length/width ratio: 0=0.5 to 1.5; 1=2 to 2.5; 2=3 to 3.3; 3=4 to 4.5.
[The puparial caudal tubercles are divided into eight types: siphonals, dorsals, dorsolaterals, laterals, ventrolaterals, ventrals, subventrals and anals. These are most likely sense organs.]
110. Caudal tubercles number: 0=3 to 4; 1=5 to 6; 2=7 to 8.
111. Siphonals: 0=absent; 1=present.
112. Dorsals: 0=absent; 1=present.
113. Dorsolaterals: 0=absent; 1=present.
114. Laterals: 0=absent; 1=present.
115. Ventrolaterals: 0=absent; 1=present.
116. Ventrals: 0=present.
117. Subventrals: 0=absent; 1=present.
118. Anals: 0=absent; 1=present.
119. Circumanalis shape: 0=demarcated; 1=elliptic or oblong; 2=fusiform or rhombic.
120. Circumanalis color: 0=black; 1=white; 2=yellow.

<u>Adult (head)</u>

121. Frons color: 0=black; 1=grey; 2=tan; 3=yellow; 4=yellowish.

[The frons is divided to different regions that may differ in their pigmentation as follows: the ocellar triangle, the frontal triangle, the region between the frontal triangle and the orbital plates, the corners behind the orbital plates, the orbital plates and the region between the frontal triangle and the ptilinal suture (Fig. S1).]

122. Frons upper color, color between the frontal triangle and the orbitals: 0=black; 1=grey; 2=tan; 3=yellow; 4=yellowish.

123. Frons lower color, color in front of the frontal triangle: 0=black; 1=grey; 2=tan; 3=white; 4=yellow; 5=yellowish.

124. Frons corners color, color behind the orbiatls: 0=black; 1=grey; 2=tan; 3=yellow; 4=yellowish.

125. Frons color pattern: 0=with patches; 1=without patches.

126. Frons shape: 0=convex; 1=flat.

127. Frons shape 2: 0=microtrichose; 1=not microtrichose.

128. Frons shininess: 0=dull; 1=shining; 2=somewhat dull.

129. Frontal length, distance from the anterior margin at the ptilinal suture to the posterior margin of the vertex in mm: 0=0.18 to 0.29; 1=0.3 to 0.37; 2=0.39 to 0.44; 3=0.49 to 0.52.

130. Frontal index, ratio between the frontal length and width just above the ptilinal suture: 0=0.57 to 0.74; 1=0.78 to 0.88; 2=0.9 to 1.1; 3=1.15 to 1.46.

131. Frontal tapering ratio, ratio of the frontal width between upper eye corners to the frontal width just above the ptilinal suture: 0=1.09 to 1.23; 1=1.26 to 1.35; 2=1.41 to 1.55.

132. Frontal triangle color: 0=black; 1=grey; 2=tan; 3=white; 4=yellow; 5=yellowish.

133. Frontal triangle shape: 0= very narrow or pointed; 1=narrow.

134. Frontal triangle width/frontal length ratio: 0=0.4 to 0.55; 1=0.6 to 0.69; 2=0.73 to 0.87.

135. Ocellar triangle height: 0=prominent; 1=slightly prominent; 2=somewhat prominent.

136. Ocellar triangle length: 0=not prolonged; 1=prolonged.

137. Ocellar triangle color: 0=black; 1=grey; 2=tan; 3=white; 4=yellow.

138. Frontal vittae: 0=absent; 1=present.

139. Frontal vittae color: 0=brown; 1=brownishyellow; 2=darkbrown; 3=darkbrownish; 4=golden-yellow.

140. Inner side of ocelli pattern: 0=with brown spots; 1=without brown spots.

141. Inner side of ocelli pilosity: 0=microtrichose; 1=not microtrichose.

142. Ocellar triangle length/frontal length ratio: 0=0.32 to 0.44; 1=24 to 38.5; 2=40 to 54.5.

143. Orbital plates color: 0=black; 1=grey; 2=white; 3=yellow; 4=yellowish.

144. Orbital plates microtrichosity: 0=microtrichose; 1=not microtrichose.

145. Orbital plates width: 0=broad; 1=indistinct; 2=narrow; 3=very broad and longer than frons.

146. Orbital plates brightness: 0=indistinct; 1=shining; 2=slightly shining; 3=subshining.

147. Orbital plates to eye margins: 0=apically divergent; 1=apically slightly divergent; 2=divergent; 3=not divergent; 4=slightly divergent.

148. Orbital plates/frontal length ratio: 0=0.42 to 0.63; 1=0.69 to 0.9; 2=1.06 to 1.08.

149. Orbital setae color: 0=black; 1=yellow.

[Orbital setae are abbreviated as follows: or1=proclinate, or2=anterior reclinate and or3=posterior reclinate]

150. Orbital setae disposition: 0=in a line or row; 1=*or2* slightly outside *or1*.

151. *or2* position: 0=*or2* behind *or1*; 1=*or2* in front of *or1*; 2=*or2* just outside *or1*.

152. *or2* width: 0=not strong; 1=strong; 2=very fine.

153. Distance between *or3* to *or1*/ distance between *or3* and medial vertical seta ratio: 0=0.43 to 1.1; 1=1.36 to 2.79; 2=68 to 68.

154. *or1*/*or3* length ratio: 0=0.56 to 0.77; 1=0.79 to 0.9; 2=0.93 to 1.18.

155. *or2*/*or1* length ratio: 0=0.32 to 0.56; 1=0.6 to 1.08.

156. Length of postocellar setae/frontal length ratio: 0=0.21 to 0.32; 1=0.48 to 0.69; 2=0.72 to 0.86.

157. Length of ocellar seta/frontal length ratio: 0=0.51 to 0.55; 1=0.62 to 0.76; 2=0.81 to 0.88; 3=0.9 to 1.1.

158. Vibrissal index, ratio in length of first genal seta/vibrissa: 0=0.21 to 0.43; 1=0.47 to 0.62; 2=0.67 to 0.83; 3=0.98 to 1.1.

159. Genal setae color: 0=black; 1=white; 2=yellowish.

160. Face color: 0=black; 1=tan; 2=white; 3=yellow; 4=yellowish.

161. Face pattern: 0=concolorous with background; 1=different colors.

162. Face microtrichosity: 0=microtrichose; 1=not microtrichose.

[The face is divided into two regions: upper region between the antennal flagellomeres I, and lower region below the antennae (Fig. S2).]

163. Carina upper length: 0=absent; 1=present.

164. Carina lower length: 0=absent; 1=present.

165. Carina upper width: 0=absent; 1=broad; 2=narrow.

166. Carina lower width: 0=absent; 1=broad; 2=divergent; 3=divergent with a distinct longitudinal groove; 4=divergent with a slight longitudinal groove; 5=narrow; 6=slightly divergent.

167. Carina upper height: 0=absent; 1=flat; 2=prominent; 3=slightly prominent.

168. Carina lower height: 0=absent; 1=bulbous; 2=flat; 3=prominent; 4=slightly prominent.

169. Carina shape: 0= not nose-like; 1=absent; 2=nose-like; 3=somewhat nose-like.

170. Cheek index, eye length/cheek width ratio: 0=13.5 to 16; 1=3 to 10.5.

171. Eye microtrichosity: 0=bare; 1=pilose.

172. Eyes shape orientation: 0=oblique; 1=perpendicular; 2=slightly oblique.

173. Eye index, eye length/eye width at right angle to length ratio: 0=1.1 to 1.14; 1=1.16 to 1.2; 2=1.21 to 1.24; 3=1.28 to 1.31.

[The occiput is divided into three regions: the margins, a medial region and the region above the foramen (Fig. S3).]

174. Occiput color: 0=black; 1=grey; 2=tan; 3=yellow; 4=yellowish.

175. Occiput marginal color 1: 0=no pattern; 1=with broad yellowish margin; 2=with narrow yellowish margin; 3=with yellowish margin.

176. Occiput pattern stripes: 0=with yellowish stripes; 1=without yellowish stripes.

177. Occiput pattern medial color: 0=concolorous; 1=darker.

178. Occiput shape: 0=concave; 1=convex.

179. Occiput color above foramen: 0=black; 1=grey; 2=tan; 3=white; 4=yellow; 5=yellowish.

180. Pedicel color: 0=black; 1=grey; 2=tan; 3=yellow; 4=yellowish.

181. Pedicel pattern: 0=concolorous; 1=dorsally darkbrown; 2=lower margin yellow; 3=with large brown spots.
182. Flagellomere 1 color: 0=black; 1=grey; 2=white; 3=yellow; 4=yellowish.
183. Flagellomere 1 shape: 0=elongate; 1=long; 2=roundish.
184. Flagellomere 1 pilosity: 0=covered with sparse and unusually long setulae; 1=not covered with unusually long setulae.
185. Arista type: 0=microtrichose; 1=plumose.
186. Arista dorsal branches: 0=0 to 0; 1=3 to 3.5; 2=4 to 5; 3=6 to 6.5.
187. Arista dorsal branches length: 0=long; 1=short.
188. Arista ventral branches: 0=0 to 1.5; 1=2 to 3; 2=6.5 to 6.5.
189. Small inner branches: 0=0 to 0; 1=5 to 8; 2=9 to 15.
190. Small inner branches length: 0=long; 1=short.
191. Arista terminal fork: 0=absent; 1=present.
192. Arista terminal fork length: 0=long; 1=short.
193. Proboscis color: 0=black; 1=white; 2=yellow; 3=yellowish.
194. Clypeus color: 0=black; 1=white; 2=yellow; 3=yellowish.
195. Palpus lower margin setae color: 0=black; 1=yellowish.
196. Palpus lower margin setae number: 0=1 to 1; 1=2 to 3; 2=4 to 6; 3=8 to 8.
197. Palpus lower margin setae length difference: 0=differentiated; 1=undifferentiated.
198. Palpus color: 0=black; 1=white; 2=yellow; 3=yellowish.

Adult (thorax)

199. Thorax length, distance from the anterior margin of the scutum to the posterior margin of the scutellum in mm: 0=0.78 to 1.08; 1=1.15 to 1.54; 2=1.77 to 2.18.
200. Scutum microtrichosity: 0=microtrichose; 1=not microtrichose.
201. Scutum microtichosity color: 0=black; 1=grey.
202. Scutum shape: 0=convex; 1=slightly flat.
[The scutum usually has a number of longitudinal colored stripes that indicate the placement of muscle attachment (Gibert et al. 2018). These stripes are divided here into a median crossing between the dorsocentral setae, two paramedians at the level of the dorsocentral setae and two laterals at the junction between the scutum and the mesopleuron.]
203. Median color stripe: 0=absent; 1=long; 2=short.
204. Paramedian color stripe: 0=absent; 1=long; 2=short.
205. Lateral color stripe: 0=absent; 1=long; 2=short.
206. Presutural setulae: 0=absent; 1=present.
207. Scutum brightness: 0=dull; 1=shining; 2=subshining.
[The scutum is divided into three colored regions as follows: the postpronotum, the prescutum and the scutum proper (Fig. S4).]
208. Postpronotum color: 0=black; 1=grey; 2=tan; 3=white; 4=yellow; 5=yellowish.
209. Prescutum color: 0=black; 1=grey; 2=tan; 3=white; 4=yellow; 5=yellowish.
210. Scutum color: 0=black; 1=grey; 2=white; 3=yellow; 4=yellowish.
211. Scutum color pattern: 0=with patches; 1=without patches.
212. Acrostichal setulae rows: 0=10 to 13; 1=2 to 4; 2=6 to 9.
213. Pospronotal seta: 0=absent; 1=present.
214. h index, upper/lower postpronotal setae length ratio: 0=0.59 to 0.98; 1=1.02 to 1.70; 2=2.35 to 4.30.

215. Transverse distance of dorsocentral setae/longitudinal distance ratio: 0=1.11 to 1.32; 1=1.65 to 2.36; 2=2.56 to 2.82; 3=4.25 to 4.27.
216. dc index, ratio in length of anterior/posterior dorsocentral setae: 0=0.53 to 0.58; 1=0.62 to 0.69; 2=0.71 to 0.81.
217. Prescutellar setae: 0=distinct or strong; 1=elongated or prolonged; 2=not well developed.
218. Scutellum shininess: 0=dull; 1=less shining; 2=subshining.
[The scutellum is divided into three colored regions as follows: the basal portion, and the lateral and apical margins.]
219. Scutellum pattern basal portion: 0=black; 1=white; 2=yellow; 3=yellowish.
220. Scutellum lateral margin: 0=black; 1=white; 2=yellow.
221. Scutellum apical margin: 0=black; 1=white; 2=yellow.
222. Scutellum median stripe: 0=absent; 1=present.
223. Scutellum pilosity: 0=bare; 1=microtrichose.
224. Distance between apical scutellar setae/distance between apical and basal setae ratio: 0=0.53 to 0.76; 1=0.78 to 1.01; 2=1.05 to 1.35.
225. Scutellar basal setae alignment: 0=convergent; 1=divergent; 2=parallel; 3=slightly convergent; 4=slightly divergent.
226. scut index, scutellar basal/apical setae length ratio: 0=0.57 to 0.72; 1=0.80 to 0.97; 2=1 to 1.23; 3=1.47 to 1.59.
[The mesopleuron is divided into two regions as follows: the upper half comprising the anepisternum and the anepimeron, and the lower half comprising the katepisternum (Fig. S5).]
227. Mesopleuron color (lower half): 0=black; 1=tan; 2=white; 3=yellow; 4=yellowish.
228. Mesopleuron color (upper half): 0=black; 1=tan; 2=white; 3=yellow; 4=yellowish.
229. Mesopleuron pattern pilosity: 0=bare; 1=microtrichose.
230. Mesopleuron pattern stripes: 0=absent; 1=present.
231. Mesopleuron pattern spots: 0=absent; 1=present.
232. Mesopleuron shininess: 0=shining; 1=slightly shining; 2=subshining.
233. sterno index, anterior/posterior katepisternal setae length ratio: 0=0.34 to 0.48; 1=0.54 to 0.58; 2=0.61 to 0.70; 3=0.75 to 0.96.
234. Median/anterior katepisternal setae length ratio: 0=0 to 0.24; 1=0.29 to 0.45; 2=0.49 to 0.73; 3=1.21 to 1.21.
235. Anterior katepisternal seta width: 0=not very fine; 1=very fine.
236. Median katepisternal seta width: 0=not very fine; 1=very fine.
237. Halter color: 0=black; 1=white; 2=yellow; 3=yellowish.
238. Coxa color: 0=black; 1=grey; 2=tan; 3=white; 4=yellow; 5=yellowish.
239. Femur color: 0=black; 1=grey; 2=tan; 3=white; 4=yellow; 5=yellowish.
240. Tibia color: 0=black; 1=grey; 2=tan; 3=white; 4=yellow; 5=yellowish.
241. Tarsomeres 1-5: 0=black; 1=grey; 2=tan; 3=white; 4=yellow; 5=yellowish.
242. Profemoral setation: 0=undifferentiated setae; 1=whit a row of prolonged anteroventral setae; 2=with 2 anteroventral rows of sharp black setae; 3=with a distinct row of 10-15 black short sharp setae.
243. Proleg tibia preapical seta: 0=absent; 1=present.
244. Proleg tibia preapical seta length: 0=long; 1=minute or small; 2=unusually long.
245. Protarsomere 1 setation: 0=sex comb; 1=undifferentiated; 2=with dense short dark setae; 3=with long fine sigmoid setae.
246. Protarsomere 1 sex comb teeth number: 0=11.5 to 12; 1=14.5 to 14.5; 2=3.5 to 4; 3=9 to 9.

247. Protarsomere 2 setation: 0=sex comb; 1=undifferentiated; 2=with dense short dark setae.
248. Protarsomere 2 sex comb teeth number: 0=13 to 13; 1=2 to 2; 2=6.5 to 9.5.
249. Protarsomere 1/protarsomere 2 length ratio: 0=almost equal; 1=protarsomere 1 distinctly longer; 2=protarsomeres 1 and 2 equal in length.
250. Sex comb teeth alignment: 0=longitudinal; 1=obliquely.
251. Mesocoxa setation: 0=with 1 strong seta; 1=with 2 strong setae; 2=without strong setae.
252. Mesotibia preapical seta: 0=absent; 1=present.
253. Mesotibia preapical seta length: 0=long; 1=minute or small.
254. Mesotibia apical seta: 0=present.
255. Mesotibia setation: 0=with prominent dorsal setae at base; 1=without prominent dorsal setae at base.
256. Metatibia preapical seta: 0=absent; 1=present.
257. Metatibia setation: 0=with a row of elongated ventral setae; 1=without elongated ventral setae.
258. Metatarsomere 1: 0=with a ventral brush; 1=without ventral brush.
259. Wing curvature in life: 0=curved down over sides of the abdomen; 1=not curved.
260. Wing vein color: 0=black; 1=grey; 2=yellow; 3=yellowish.
261. Costa length: 0=ending at R_{4+5}; 1=ending before R_{4+5}.
262. CI vein color: 0=dark brown; 1=diffuse shadow; 2=hyaline; 3=with a faint narrow shadow.
263. CI vein tip color: 0=black; 1=dark brown; 2=diffuse shadow; 3=hyaline; 4=pale; 5=with a faint narrow shadow.
264. Second costal break length: 0=deep; 1=shallow.
265. CII and CIII veins color: 0=darkbrown; 1=diffuse shadow; 2=hyaline; 3=with a faint narrow shadow.
266. CIII costal pegs: 0=absent; 1=present.
267. Subcosta/R_1 veins connection: 0=fused; 1=separated.
268. R_1 radial vein tip color: 0=dark; 1=hyaline.
269. R_{2+3} radial vein before tip color: 0=dark; 1=hyaline.
270. R_{2+3} radial vein tip color: 0=dark spot; 1=hyaline; 2=slightly darkened and shadowed.
271. R_{4+5} radial vein tip color: 0=dark spot; 1=hyaline; 2=slightly darkened and shadowed.
272. Crossveins color: 0=black; 1=grey; 2=yellow.
273. bM-Cu crossvein: 0=absent; 1=present.
274. R-M crossvein color: 0=dark; 1=yellow.
275. dM-Cu crossvein band: 0=absent; 1=present.
276. Anal cell and A_1 vein: 0=absent; 1=present.
277. Veins R_{4+5} and M alignment: 0=apically distinctly convergent; 1=parallel; 2=slightly convergent; 3=strongly convergent.
278. CIV/CIII thickness ratio: 0=same thickness; 1=thinner.
279. Wing length, distance between basal bifurcation of M and wing tip: 0=1.9 to 2.44; 1=2.53 to 3.31; 2=3.61 to 3.96.
280. Wing length/width length ratio, wing width being the maximum width usually between R-M and dM-Cu crossveins: 0=2.05 to 2.1; 1=2.14 to 2.2; 2=2.22 to 2.29; 3=2.34 to 2.46.
[Wing shape is summarized in terms of eight indices (Fig. S6).]

281. C index, CII/CIII veins length ratio: 0=1.43 to 2.43; 1=2.56 to 2.9; 2=3.06 to 3.43; 3=3.66 to 4.37.
282. ac index, CIII/CIV veins length ratio: 0=1.33 to 4.47; 1=9.27 to 9.27.
283. hb index, CIII with heavy bristles/total CIII length ratio: 0=0.23 to 0.39; 1=0.4 to 0.58; 2=0.67 to 0.78.
284. 4C index, CIII/MIII length ratio: 0=0.49 to 0.96; 1=1.03 to 1.43; 2=1.66 to 1.84; 3=2.62 to 2.62.
285. 4v index, MIV/MIII length ratio: 0=1.23 to 1.72; 1=1.83 to 3.05; 2=4.37 to 4.63.
286. 5x index, CuA (apical section)/dM-Cu length ratio: 0=1.03 to 1.27; 1=1.35 to 2.42; 2=3.68 to 3.68; 3=6.77 to 6.77.
287. M index, CuA (apical section)/MIII length ratio: 0=0.32 to 0.62; 1=0.66 to 0.93; 2=1.92 to 2.28.
288. prox.x index, basal R_{4+5}/MIII length ratio: 0=0.4 to 1.05; 1=1.17 to 1.79.

Adult (abdomen)

[The abdomen is usually patterned having both light and dark colors. This difference was conceptualized as two characters. However, when the abdomen was unicolorous, such as "entirely black", the two characters had the same state.]

289. Light ground color: 0=black; 1=grey; 2=white; 3=yellow; 4=yellowish.
290. Dark ground color: 0=black; 1=grey; 2=white; 3=yellow.

[The male abdomen has five visible tergites denoted A2-A6. Tergites usually have symmetrical color patterns. To code for the patterns, each tergite (right half) was divided into six regions according to the antero-posterior and the proximo-distal axes (Fig. S7).]

291. A2 anterodistal color: 0=dark; 1=light; 2=partially dark.
292. A2 anteromedial color: 0=dark; 1=light; 2=partially dark.
293. A2 anteroproximal color: 0=dark; 1=light; 2=partially dark.
294. A2 posterodistal color: 0=dark; 1=light; 2=partially dark.
295. A2 posteromedial color: 0=dark; 1=light; 2=partially dark.
296. A2 posteroproximal color: 0=dark; 1=light; 2=partially dark.
297. A2 shininess: 0=shining; 1=subshining.
298. A3 anterodistal color: 0=dark; 1=light; 2=partially dark.
299. A3 anteromedial color: 0=dark; 1=light; 2=partially dark.
300. A3 anteroproximal color: 0=dark; 1=light; 2=partially dark.
301. A3 posterodistal color: 0=dark; 1=light; 2=partially dark.
302. A3 posteromedial color: 0=dark; 1=light; 2=partially dark.
303. A3 posteroproximal color: 0=dark; 1=light; 2=partially dark.
304. A3 shininess: 0=shining; 1=subshining.
305. A4 anterodistal color: 0=dark; 1=light; 2=partially dark.
306. A4 anteromedial color: 0=dark; 1=light; 2=partially dark.
307. A4 anteroproximal color: 0=dark; 1=light; 2=partially dark.
308. A4 posterodistal color: 0=dark; 1=light; 2=partially dark.
309. A4 posteromedial color: 0=dark; 1=light; 2=partially dark.
310. A4 posteroproximal color: 0=dark; 1=light; 2=partially dark.
311. A4 shininess: 0=shining; 1=subshining.
312. A4 lateroventral expansion: 0=absent; 1=present.
313. A5 anterodistal color: 0=dark; 1=light; 2=partially dark.
314. A5 anteromedial color: 0=dark; 1=light; 2=partially dark.
315. A5 anteroproximal color: 0=dark; 1=light; 2=partially dark.

316. A5 posterodistal color: 0=dark; 1=light; 2=partially dark.
317. A5 posteromedial color: 0=dark; 1=light; 2=partially dark.
318. A5 posteroproximal color: 0=dark; 1=light; 2=partially dark.
319. A5 shininess: 0=shining; 1=subshining.
320. A5 lateroventral expansion: 0=absent; 1=present.
321. A6 anterodistal color: 0=dark; 1=light; 2=partially dark.
322. A6 anteromedial color: 0=dark; 1=light; 2=partially dark.
323. A6 anteroproximal color: 0=dark; 1=light; 2=partially dark.
324. A6 posterodistal color: 0=dark; 1=light; 2=partially dark.
325. A6 posteromedial color: 0=dark; 1=light; 2=partially dark.
326. A6 posteroproximal color: 0=dark; 1=light; 2=partially dark.
327. A6 shininess: 0=shining; 1=subshining.
328. A6 dorsal width: 0=as broad as long; 1=shorter.

Adult (male terminalia)
329. Epandrial phragma dorsal width: 0=absent; 1=broad; 2=narrow.
330. Epandrial phragma ventral width: 0=absent; 1=broad; 2=narrow.
331. Epandrial anteroventral expansion: 0=distinct; 1=indistinct; 2=protruding.
332. Epandrial microtrichosity: 0=absent; 1=mostly microtrichose; 2=partially microtrichose.
333. Epandrial texture: 0=medially slightly rugose; 1=not rugose.
334. Epandrial dorsal connection: 0=constricted; 1=contiguous; 2=membranous.
335. Epandrial lower setae: 0=0 to 2; 1=15 to 23; 2=32 to 37; 3=6 to 12.
336. Epandrial lower setae type: 0=prensisetae; 1=trichoid.
337. Epandrial upper setae: 0=0 to 3; 1=10 to 18; 2=38 to 38; 3=5 to 9.
338. Epandrial ventral lobe: 0=absent; 1=covering surstylus; 2=not covering surstylus; 3=partly covering surstylus; 4=protruding beyond surstylus.
339. Epandrial ventral lobe microtrichosity: 0=absent; 1=mostly microtrichose; 2=partially microtrichose.
340. Epandrial posteroventral margin shape: 0=expanded; 1=lobate; 2=straight.
341. Epandrial posteroventral margin sclerotization: 0=expanded; 1=membranous; 2=partially membranous; 3=sclerotized.
342. Epandrial ventral lobe texture: 0=dorsally slightly rugose; 1=mostly rugose; 2=not rugose.
343. Epandrial posterior lobe size: 0=large; 1=small; 2=very large.
344. Epandrial posterior lobe shape: 0=blade-shaped, apically blunt and serrate; 1=distally rounded; 2=not protruding; 3=square.
345. Epandrial posterior lobe texture: 0=covered with tiny scales; 1=not covered with scales.
346. Cercus connection to epandrium: 0=by membranous tissue; 1=fused; 2=partially fused.
347. Cercal microtrichosity: 0=absent; 1=fully microtrichose; 2=mostly microtrichose; 3=partially microtrichose.
348. Cercal anteroventral expansion: 0=absent; 1=present.
349. Cercal anterodorsal expansion: 0=absent; 1=present.
350. Cercal lateral expansion: 0=absent; 1=present.
351. Cercal ventral connection: 0=absent; 1=present.
352. Cercal ventral lobe: 0=absent; 1=present.

353. Cercal ventral lobe protrusion: 0=narrow, remarkably convergent; 1=not protruding; 2=protruding inward.
354. Cercal peg-like setae: 0=0 to 0; 1=12 to 15; 2=2 to 3.
355. Surstylus: 0=absent; 1=present.
356. Surstylar microtrichosity: 0=absent; 1=fully microtrichose; 2=mostly microtrichose; 3=partially microtrichose.
357. Surstylar prensisetae alignment: 0=concave; 1=convex; 2=slightly concave; 3=slightly convex; 4=straight.
358. Surstylar prensisetae lateral expansion: 0=with 2 lateral isolated peg-like setae; 1=without isolated setae.
359. Surstylar prensisetae number: 0=0 to 1; 1=14 to 21; 2=31 to 31; 3=5 to 13.
360. Surstylar inner setae: 0=0 to 2; 1=24 to 28; 2=54 to 54; 3=6 to 16.
361. Surstylar outer setae: 0=0 to 2; 1=18 to 19; 2=3 to 10.
362. Surstylar outer setae type: 0=prensisetae; 1=trichoid.
363. Surstylar anterodorsal lobe: 0=absent; 1=membranous; 2=protruding; 3=sclerotized; 4=strongly sclerotized.
364. Surstylar connection to epandrium: 0=by membranous tissue; 1=fused; 2=partially fused.
365. Decasternum: 0=neither narrow nor well developed; 1=very narrow or reduced; 2=well developed.
366. Decasternum shape: 0=neither triangular nor rectangular; 1=rectangular; 2=triangular.
367. Decasternum lateral sclerotization: 0=membranous; 1=strongly sclerotized; 2=weakly sclerotized.
368. Decasternum distal margin: 0=medially notched; 1=not medially notched.
369. Decasternum connection to epandrium: 0=by a long sclerotized narrow strip; 1=by membranous tissue.
370. Decasternal dorsal arch: 0=absent; 1=developed; 2=well developed.
371. Decasternal dorsal arch to aedeagal apodeme: 0=parallel; 1=perpendicular.
372. Decasternal dorsal arch ventral expansion: 0=backwards; 1=forwards; 2=no expansion.
373. Decasternal dorsal arch layers: 0=no layers; 1=three.
374. Decasternal dorsal arch texture: 0=scales; 1=smooth.
375. Decasternal dorsal arch scales: 0=blunt-tipped; 1=not blunt-tipped.
376. Decasternal dorsl arch smarginal sclerotization: 0=membranous; 1=sclerotized.
377. Decasternal microtrichosity: 0=absent; 1=partially microtrichose.
378. Decasternal position: 0=horizontally positioned; 1=neither horizontally nor upper; 2=upper positioned.
379. Hypandrium/epandrium length ratio: 0=as long as; 1=longer; 2=shorter.
380. Hypandrial lateral margin: 0=expanded anteriorly and dorsoposteriorly; 1=expanded dorsoanteriorly; 2=expanded dorsoposteriorly; 3=slightly expanded posteriorly; 4=slightly sinuate; 5=straight.
381. Hypandrial anterior margin: 0=concave; 1=convex; 2=narrow; 3=straight.
382. Hypandrial posterior process: 0=absent; 1=present.
383. Hypandrium to aedeagus alignment: 0=parallel; 1=perpendicular.
384. Gonopod ventral to dorsal halves: 0=linear; 1=perpendicular.
385. Gonopod texture: 0=not rugose; 1=submedially slightly rugose.

386. Gonopod dorsal branch shape: 0=absent; 1=bifurcate; 2=bifurcate broad; 3=bifurcate median protruding; 4=bifurcate median protruding fused with ventral branch; 5=bifurcate narrow anterad; 6=slightly bifurcate; 7=truncate.
387. Gonopod dorsal connection to each other: 0=absent; 1=broad concave process; 2=straight; 3=thin concave process complete; 4=thin concave process incomplete; 5=thin convex process.
388. Gonopod dorsal connection (thin convex process): 0=not ribbon-shaped; 1=ribbon-schaped.
389. Gonopod dorsal connection (broad concave process): 0=not backwardly expanded; 1=well-developed and backwardly expanded.
390. Gonopod dorsal connection (broad concave process) asymmetry: 0=not asymmetric; 1=slightly asymmetric; 2=strongly asymmetric.
391. Gonopod dorsal connection (thin concave process complete) sclerotization: 0=membranous; 1=sclerotized.
392. Gonopod ventral connection to hypandrium: 0=by membranous tissue; 1=fully fused; 2=partially fused.
393. Gonopod ventral connection to each other: 0=fused; 1=separated.
394. Gonopod ventral setae and setulae: 0=0; 1=1 to 3; 2=27 to 27; 3=9 to 11.
395. Gonopod microtrichosity: 0=absent; 1=fully microtrichose; 2=mostly microtrichose; 3=partially microtrichose.
396. Aedeagus: 0=absent; 1=present.
397. Aedeagus connection to apodeme: 0=by membranous tissue; 1=fused; 2=partially fused.
398. Aedeagus dorsoventral curvature, angle between the line connecting the tip of the aedeagus to the mid of the aedeagus and the aedeagal apodeme axis: 0=141 to 193; 1=203 to 221; 2=60 to 127.
399. Aedeagus basal curvature: 0=bent; 1=straight; 2=strongly bent.
400. Aedeagus submedially curvature: 0=slightly bent dorsad; 1=straight.
401. Aedeagus apical curvature: 0=abruptly curved; 1=sinuate; 2=slightly sinuate; 3=straight.
402. Aedeagus ventroapical curvature: 0=bent ventrad; 1=straight.
403. Aedeagus dorsoapical curvature: 0=protruding dorsad; 1=shaped like a telephone receiver; 2=sharply pointed dorsad; 3=straight.
404. Aedeagus length, distance from the basal connection with the aedeagal apodeme to the apical tip in mm: 0=0.12 to 0.23; 1=0.25 to 0.45; 2=0.57 to 0.77.
405. Aedeagus width, largest width of the aedeagus in mm: 0=0.021 to 0.055; 1=0.058 to 0.078; 2=0.094 to 0.152.
406. Aedeagus basal/apical width ratio: 0=broader; 1=equal; 2=much broader; 3=narrower; 4=slightly narrower.
407. Aedeagus ventral expansion: 0=expanded; 1=not expanded; 2=remarkably expanded; 3=slightly expanded.
408. Aedeagus dorsal expansion: 0=expanded; 1=not expanded.
409. Aedeagus lateral expansion: 0=expanded; 1=not expanded; 2=slightly expanded.
410. Aedeagus medial width: 0=expanded; 1=not expanded.
411. Aedeagus submedial width: 0=expanded dorsoventrally; 1=not expanded.
412. Aedeagus subapical width: 0=anteriorly expanded; 1=not expanded; 2=slightly expanded.
413. Aedeagus apical width: 0=expanded; 1=narrowed; 2=not narrowed or expanded; 3=pointed; 4=slightly expanded; 5=slightly narrowed.

414. Aedeagus lateral texture: 0=mostly rugose, each wrinkle ending as a tiny scale; 1=reticulate recalling the chorion of a drosophilid egg; 2=smooth; 3=with a fringe-like irregular row of long and blunt scales; 4=with tiny scales.
415. Aedeagus microtrichosity: 0=covered with thin and reltively dense microtrichia; 1=dorsolaterally slightly microtrichose in median area; 2=not microtrichose; 3=ventrally microtrichose; 4=ventrally slightly microtrichose.
416. Aedeagus basal forward protrusion: 0=not protruding; 1=slightly protruding forwards.
417. Aedeagus dorsal cleft size: 0=1/4 of aedeagus size; 1=3/4 of aedeagus size; 2=half of aedeagus length; 3=small.
418. Aedeagus dorsal cleft membrane: 0=with a loose membrane, expanded dorsad and as broad as base of aedeagus; 1=with no membrane.
419. Aedeagus dorsal cleft lateroanterad projection: 0=not projecting anterad; 1=projecting anterad.
420. Aedeagus basal sclerotization: 0=membranous; 1=sclerotized; 2=strongly sclerotized.
421. Aedeagus apical sclerotization: 0=membranous; 1=sclerotized; 2=slightly membranous.
422. Aedeagus dorsomedioapical sclerotization: 0=membranous; 1=sclerotized; 2=slightly membranous.
423. Aedeagus dorsosubmedial texture: 0=smooth; 1=with a fringed collar of scales; 2=with scales; 3=with tiny scales.
424. Aedeagus ventrosubmedial texture: 0=smooth; 1=with a fringed collar of scales; 2=with scales; 3=with tiny scales.
425. Aedeagus apicomarginal texture: 0=margin with tiny scales; 1=marginally serrate; 2=marginally subtly serrate; 3=with no serrations or scales.
426. Aedeagus ventrodistal texture: 0=covered with scales; 1=with no scales.
427. Aedeagus dorsodistal texture: 0=marginally finely serrate; 1=marginally serrate; 2=serrate; 3=with no serrations.
428. Aedeagus dorsosubmedial dorsal protrusion: 0=with a dorsal process, roundish-tipped in lateral view; 1=with no protrusion.
429. Aedeagus dorsomedial dorsal projection: 0=with a large asymmetric anterad projecting branched process; 1=with no projection.
430. Aedeagus mediolateral texture: 0=with a fusion line along its length; 1=with no fusion line.
431. Aedeagus subapical texture: 0=marginally serrate dorsally; 1=with no serration.
432. Aedeagus ventrosubapical protrusions: 0=with a pair of long, distally slightly divergent ventral processes, preceded by a pair of outer, small, curved, posterad projecting processes; 1=with a pair of long, ventrally directed processes which converge at tip, and distally slightly encircle paraphyses; 2=with no protrusions.
433. Aedeagus dorsosubapical protrusions: 0=with a pair long slightly bent subapical dorsad projecting processes margin; 1=with a pair of long dorsally projecting processes which are covered with tiny scales, anteriorly pointed, slightly sinuate process, and apically partially encircled by paraphyses; 2=with a pair of tiny, dorsad directed dorsal processes, preceded by one tiny dorsad curved projection; 3=with no protrusions.
434. Aedeagus dorsoapical protrusion: 0=strongly bifurcate with two thin horn-shaped branches which are covered with tiny scales and bent inwards and dorsad; 1=with a pair of sharply pointed, small processes which are projected posterad in lateral

and slightly divergent in ventral view; 2=with a pair of thin dorsad directed projections; 3=with no protrusions.

435. Aedeagus ventrodistal protrusion: 0=no protrusion; 1=projecting backwards; 2=with a V-shaped process encircling gonopore.

436. Aedeagus apical shape: 0=blunt and slightly bifid at tip; 1=dorsodistally blunt; 2=folded over itself, reaching ventral margin of aedeagal apodeme; 3=roundish.

437. Aedeagus apical asymmetry: 0=somewhat asymmetric; 1=symmetric.

438. Inner paraphysis branches: 0=absent; 1=branched; 2=trifurcate; 3=unbranched.

439. Inner paraphyses sclerotization: 0=anteriorly and posteriorly strongly sclerotized but weakly sclerotized in median region; 1=anterolaterally membranous and posteriorly sclerotised; 2=sclerotized; 3=strongly sclerotized.

440. Inner paraphyses tip (in unbranched species): 0=expanded laterally; 1=pointed; 2=roundish; 3=with a finger-shaped, curved, backwardly-directed process.

441. Inner paraphyses dorsal shape (in branched species): 0=not sinuate; 1=sinuate.

442. Inner paraphyses dorsal tip (in branched species): 0=not sharply pointed; 1=sharply pointed.

443. Inner paraphyses ventral branch size (in trifurcate species): 0=broad; 1=narrow.

444. Inner paraphyses median branch texture (in trifurcate species): 0=not similar to gooseflesh; 1=similar to gooseflesh.

445. Inner paraphyses median branch size (in trifurcate species): 0=dorsoventrally flattened; 1=well-developed.

446. Inner paraphyses median branch tip shape (in trifurcate species): 0=hook-shaped; 1=not pointed.

447. Inner paraphyses dorsal branch texture (in trifurcate species): 0=covered with tiny scales; 1=not covered with tiny scales.

448. Inner paraphyses dorsal branch size (in trifurcate species): 0=dorsoventrally flattened; 1=narrow.

449. Inner paraphyses dorsal branch tip shape (in trifurcate species): 0=blunt; 1=pointed.

450. Inner paraphyses connection: 0=anterodorsally fused to each other; 1=fused to each other dorsally; 2=inner margins fused to each other and forming a keel-shaped blade perpendicular to adeagus and situated between paraphyses.

451. Outer paraphyses shape: 0=absent; 1=bifurcate; 2=rod bent dorsally; 3=sinuate; 4=spatulate; 5=trifurcate.

452. Outer paraphyses longer branch (in trifurcate species): 0=dorsal longer; 1=median longer; 2=ventral longer.

453. Outer paraphyses ventral tip shape: 0=blunt; 1=broad; 2=pointed; 3=posteriorly widely expanded outwards; 4=rounded; 5=submedially expanded dorsad, 4x longer than wide.

454. Outer paraphyses connection to gonopods: 0=apposed; 1=by membranous tissue; 2=fused; 3=partially fused.

455. Outer paraphyses connestion: 0=proximally fused to each other beneath aedeagus; 1=separate.

456. Outer paraphyses setulae alignment: 0=parallel; 1=perpendicular.

457. Outer paraphyses microtrichosity: 0=absent; 1=partially microtrichose.

458. Outer paraphyses peg-like setae number: 0=absent; 1=present.

459. Outer paraphyses trichoid setae number: 0=absent; 1=present.

460. Outer paraphyses setulae number: 0=0 to 1; 1=2 to 5; 2=6 to 10.

461. Outer paraphyses setulae position: 0=apically; 1=laterally; 2=subapically.

462. Outer paraphyses connection to aedeagal apodeme: 0=broadly fused to ventral rod; 1=by membranous tissue; 2=by membranous tissue (to ventral rod).
463. Aedeagal apodeme/aedeagus length ratio: 0=0.25 to 0.5; 1=0.75 to 0.75; 2=1 to 1.25; 3=2 to 2.
464. Aedeagal apodeme shape: 0=bent; 1=bifurcate; 2=rod.
465. Aedeagal apodeme anterior half in lateral view: 0=expanded; 1=thin.
466. Aedeagal apodeme anterior half in ventral view: 0=bifid; 1=expanded; 2=plate-like; 3=thin.
467. Aedeagal apodeme posterior shape: 0=bifid; 1=not bifid.
468. Aedeagal apodeme to hypandrial arms connection: 0=fused; 1=separate.
469. Ventral rod: 0=absent; 1=present; 2=vestigial.
470. Ventral rod shape: 0=positioned apically; 1=positioned medially.
471. Ventral rod to aedeagus alignment: 0=parallel to aedeagus; 1=perpendicular to aedeagus.
472. Ventral rod protrusion: 0=medially slightly projected dorsad; 1=not protruding; 2=protruding ventrad beyond hypandrium.
473. Ventral rod texture: 0=not grooved; 1=slightly grooved medially.
474. Ventral rod shape: 0=distally expanded and dorsoventrally flattened; 1=dorsoventrally flattened; 2=not expanded.
475. Ventral rod sclerotization: 0=conspicuously ribbon-shaped weakly sclerotized very long and flexible; 1=sclerotized.
476. Ventral rod microtrichosity: 0=absent; 1=microtrichose.
477. Ventral rod/aedeagal apodeme length ratio: 0=0.5 to 1; 1=1.5 to 2; 2=4 to 4; 3=6 to 6.
478. Ventral rod connection to hypandrium: 0=by membranous tissue; 1=fused; 2=weakly fused to hypandrium.

Adult (female terminalia)
479. Cerci: 0=absent; 1=present.
480. Tergite 8 sclerotized plates: 0=absent; 1=present.
481. Oviscapt: 0=absent; 1=present.
482. Oviscapt bristles: 0=peg-like; 1=trichoid.
483. Oviscapt dorsal shape: 0=almost straight; 1=expanded dorsally; 2=rounded.
484. Oviscapt ventral shape: 0=almost straight; 1=rounded; 2=slightly rounded.
485. Oviscapt isthmus: 0=broad; 1=narrow.
486. Oviscapt discal pegs: 0=0 to 3; 1=11 to 13; 2=23 to 27; 3=4 to 7.5.
487. Oviscapt marginal pegs: 0=14.5 to 18; 1=19 to 24; 2=2 to 2; 3=9 to 12.5.
488. Oviscapt peg shape at tip: 0=mostly roundish; 1=roundish; 2=sharp.
489. Inner subterminal ovisensilla length: 0=long; 1=short; 2=very long.
490. Inner subterminal ovisensilla curvature: 0=curved; 1=slightly curved; 2=straight.
